# A digital marker for stratifying cardiovascular metabolic comorbidities among the middle-aged and elderly adults

**DOI:** 10.1371/journal.pdig.0001528

**Published:** 2026-07-02

**Authors:** Danhui Mao, Sheng Zhao, Jiao Lu

**Affiliations:** 1 Shanxi Medical University, Taiyuan, China; 2 Shanxi Bethune Hospital, Shanxi Academy of Medical Sciences, Third Hospital of Shanxi Medical University, Tongji Shanxi Hospital, Taiyuan, China; 3 School of Public Policy and Administration, Xi’an Jiaotong University, Xi’an, China; Liverpool John Moores University - City Campus: Liverpool John Moores University, UNITED KINGDOM OF GREAT BRITAIN AND NORTHERN IRELAND

## Abstract

Cardiovascular Metabolic Comorbidities (CMM) share common physiological mechanisms in inflammation and immunity, oxidative stress, and insulin resistance, leading to mutual disease interactions and complex clinical manifestations. To address challenges in describing CMM status based solely on clinical features, this paper proposes a digital marker to characterize the differences from single to multiple diseases, systematically revealing distinct CMM subgroups based on cross-sectional data. This paper constructed a directed acyclic network for CMM using demographic characteristics, clinical laboratory parameters, and disease status as nodes via the *DirectLiNGAM* algorithm. Network features were described using in degree, out degree, degree centrality, betweenness centrality, and closeness centrality, ranking node importance. The top seven significant clinical laboratory parameters were selected based on this ranking. Subsequently, the performance of ten machine learning algorithms (*Random Forest*, *XGBoost*, *MLP*, *KNN*, *Gradient Boosting*, *SVC*, *Linear Regression*, *Ridge*, *ElasticNet*, *Lasso*) in generating digital markers by predicting death was evaluated to determine the optimal algorithm. The generated digital markers were then binned to classify CMM into Low, Middle, and High groups. Finally, linear regression validated the rationality of the network filtered clinical laboratory parameters. In the CMM network, the top three disease nodes by in degree are DM, MemD, and DL, while the top five by out degree are TC, HBALC, GLU, HCT, and HGB. Regarding network centrality, the top five nodes by degree centrality are Male, TG, DM, CYC, and DL; by betweenness centrality, Male, Stroke, TG, DL,and DM; and by closeness centrality, Male, DM, Married, Stroke, and CA. Network analysis identified top clinical laboratory parameters as GLU, HBALC, TC, UA, HCT, TG, HGB, WBC, and CYC, consistent with statistically significant parameters (*P* < 0.05) in linear regression validation. Among machine learning algorithms, *Ridge regression* performed best in *AUC*, *PR‑AUC*, *Brier Score*, and *Log Loss*. The digital marker generated by *Ridge regression* yielded average scores of 0.016(0.008), 0.058(0.019), 0.296(0.197) for Low, Middle, and High groups, respectively. This paper developed a digital marker by integrating network analysis and machine learning to delineate CMM’s cross‑sectional subgroups, indicating its potential for early detection and enabling future research on stratified interventions for high risk groups.

## Author summary

Cardiovascular Metabolic Comorbidities (CMM) involve multiple interrelated diseases, including diabetes, heart disease, and kidney disease, which share common biological pathways such as inflammation, oxidative stress, and insulin resistance. In clinical practice, patients often present with multiple complex and overlapping conditions, which require physicians to adopt a holistic approach and provide systematic, coordinated, and comprehensive management grounded in general practice principles. However, current clinical practice still lacks a simple and quantifiable indicator to assess patients’ overall CMM burden, nor does it effectively stratify individuals along the continuum from single to multiple diseases. This poses a significant challenge for identifying high-risk populations and developing personalized intervention strategies in general practice settings. To address this gap, the present study aims to break through the limitations of traditional single-disease diagnostic frameworks and explore a novel digital marker that integrates routine blood test parameters and demographic information. Guided by the principles of comprehensiveness and continuity of care in general practice, we are committed to achieving continuous quantitative assessment and subgroup classification of CMM burden, thereby providing a practical tool for the health management of older adults in general practice settings.

## 1. Introduction

Cardiovascular metabolic comorbidities (CMM) represent a complex disease cluster involving multiple interacting pathophysiological mechanisms, including inflammation, oxidative stress, insulin resistance, vascular calcification, mitochondrial dysfunction, fibrosis, and interstitial injury [1–7], which mutually reinforce each other and lead to clinical manifestations that are difficult to explain with single-disease models. To date, epidemiological studies on CMM have predominantly focused on older adult populations and developed countries [8]. Compared to patients with a single cardiovascular or metabolic disease, individuals with CMM face more severe health challenges, including a significant decline in quality of life and physical function, markedly increased hospitalization and mortality rates, and a substantial disease burden on healthcare systems [9,10]. Since the progression of CMM constitutes a continuous process, stratifying its cross-sectional subgroups across diverse populations is essential for early identification and intervention, forming a core component of effective prevention and management strategies [11].

Early epidemiological studies on CMM predominantly described prevalence and risk factors using traditional regression-based approaches [12]. As the understanding of multimorbidity evolved, researchers began to identify stable disease clusters, with CMM recognized as one of the most consistent patterns [13,14]. However, these phenotyping studies often lacked a thorough exploration of complex interrelationships, such as mediating, moderating, and interactive effects, among demographic characteristics, clinical laboratory parameters, and disease statuses [15–18]. More recently, complex network analysis has been employed to better characterize associations between diseases [19]. For instance, Wang et al. applied network analysis to classify chronic disease clusters incorporating demographic characteristics [20]. In our previous work, we utilized network analysis to categorize combinations of five cardiovascular or metabolic diseases (hypertension, dyslipidemia, diabetes, CKD, and hyperuricemia) while more comprehensively considering clinical laboratory parameters [21].

Although some progress has been made in previous studies, a critical limitation remains. Existing research either fails to adequately incorporate clinical laboratory parameters, covers a limited range of disease types, or remains confined to the classification of disease clusters. It does not achieve quantitative subgroup stratification within the multimorbidity network. Given the shared pathophysiological mechanisms among CMM diseases, these mechanisms cause the clinical laboratory parameters of individual conditions to become interconnected. They also interfere with one another in the context of multimorbidity. Consequently, traditional diagnostic frameworks for single diseases are inherently insufficient. Staging criteria that rely on a limited number of indicators also fail. They cannot capture the complex interactions in multimorbid states. Specifically, three major gaps persist. First, the interactive effects among clinical laboratory parameters (e.g., glycated hemoglobin A1c, fasting blood glucose, hematocrit, etc.) have not been systematically modeled. Second, the interplay between demographic characteristics (e.g., age, gender, etc.) and laboratory parameters is often overlooked. Third, the network structure linking multiple chronic diseases lacks quantitative subgroup stratification. As the understanding of the complexity of CMM continues to deepen, there is an urgent need for a new approach. This approach should integrate multidimensional clinical laboratory parameters and demographic characteristics to generate a quantifiable marker. Such an approach would enable continuous assessment of multimorbidity burden and subgroup stratification in CMM patients. It would thereby provide clinical decision support for identifying high-risk individuals and developing personalized intervention strategies.

Building upon the common CMM conditions examined in previous research (Hypertension, Diabetes Mellitus, Heart Disease, Stroke, Dyslipidemia, Chronic Kidney Disease) and further incorporating other relevant chronic diseases (Cancer, Chronic Pulmonary Diseases, Mental Disorders, Arthritis, Liver Diseases, Gastrointestinal Diseases, Asthma, and Memory Disorders) [22–28], this paper proposes a methodological framework that combines network analysis and machine learning to generate a digital marker for stratifying CMM subgroups. The research hypothesis of this paper is that by screening and integrating key clinical laboratory features and demographic characteristics, a digital marker can be constructed to achieve subgroup classification of CMM, thereby providing a quantifiable multimorbidity assessment tool for clinical practice and assisting in the disease management of CMM. Specifically, this paper accomplished the following: ① A structural network for CMM was constructed to effectively represent the complex relationships among disease statuses, demographic characteristics, and clinical laboratory parameters. ② Through network characteristic analysis, key clinical laboratory parameters suitable for stratifying CMM progression were identified. ③ The machine learning algorithms were compared. By integrating key clinical laboratory parameters and demographic characteristics to predict the total number of disease types, a digital marker was generated. This biomarker was subsequently partitioned to define the subgroups of CMM. ④ Finally, a statistical description of demographic characteristics and clinical laboratory tests was performed across different CMM subgroups. The reliability of the selected clinical laboratory parameters was further validated.

## 2. Methods

### 2.1. Patients and study design

**Fig 1:**
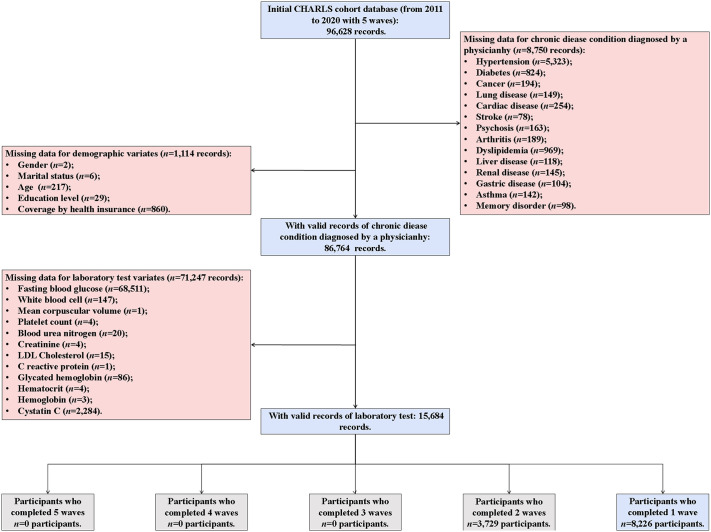
Flow diagram of the screening of the participants.

This paper selected participants from the China Health and Retirement Longitudinal Study (CHARLS) conducted between 2011 and 2020 as the research subjects. The detailed design methodology of CHARLS is available through the public website at https://charls.pku.edu.cn/. During the data preprocessing stage, we first rigorously cleaned samples with missing values to ensure the completeness and reliability of demographic characteristics, clinical laboratory parameters, and disease status data. Subsequently, we conducted further screening to exclude individuals who had completed only one survey waves. See more details in Fig 1. After the aforementioned screening process, a total of 8,226 individuals were included in this paper, and their detailed are summarized and presented in Table 2. The fieldwork for each household questionnaire and the biomarker collection was approved. This paper protocol was reviewed and approved by the Ethics Committee of Shanxi Medical University (Approval No: 2025GLL033), and the study procedures strictly adhered to the principles of the Declaration of Helsinki. Furthermore, the CHARLS project itself has obtained ethical approval from the Ethics Review Committee of Peking University (Approval No. for household surveys: IRB00001052‑11015; Approval No. for biomarkers collection: IRB00001052‑11014). All survey participants provided written informed consent.

### 2.2. Measurements of diseases status, clinical laboratory parameters and demographic characteristics

This paper included 14 chronic conditions for analysis: hypertension, diabetes, heart disease, stroke, dyslipidemia, chronic kidney disease, cancer, chronic lung disease, mental disorders, arthritis, liver disease, gastrointestinal disease, asthma, and memory disorders. The presence of each chronic condition was determined based on self-reported physician diagnosis in the CHARLS questionnaire, which records whether a participant had ever been diagnosed with the condition by a doctor. The diagnostic information reflects cumulative prevalence up to the survey year and was used to determine the overall multimorbidity burden during the study period. The collection of blood and urine samples from participants after a prescribed fasting period was performed by the survey team. Following on-site preliminary processing, these samples were consistently transported under controlled low-temperature conditions to a designated central laboratory. Subsequently, all biochemical analyses were conducted by certified laboratory technicians using standardized methodologies within the accredited facility. The demographic characteristics involved in the paper include Age, gender, marital status, education level, living area. See more details in Table 2.

### 2.3. CMM network construction and test characteristics selection

Prior to network construction, continuous variables were standardized using Z-score normalization to ensure comparability of scales. Categorical variables were encoded using one-hot encoding. The structural network was constructed using demographic characteristics, clinical laboratory parameters, and disease status as nodes. The structural relationships among variables were identified using the *DirectLiNGAM* algorithm (Linear Non-Gaussian Acyclic Model), and edges were established to build the CMM network [29]. The *DirectLiNGAM* algorithm employs an iterative procedure to directly determine the structural order among variables. In this work, the direction of edges between nodes in the *DirectLiNGAM* algorithm was determined by iterating the following two steps. First, for each variable *X*_*j*_, a linear regression of *X*_*j*_ on all other variables *X*_*-j*_ was performed to obtain the residual *r* ^*(j)*^, as specified in Equation 1. The independence between *X*_*j*_ and the residual vector *r* ^*(j)*^ was then calculated, and the variable with the highest independence was identified as the root node. Second, all other variables were regressed on this root node to obtain regression residuals, forming a new residual dataset with the influence of the root node removed. Within this new residual dataset, the step of identifying the root node was repeated to determine the next root node. These steps were iterated until the structural order of all variables was established. The causal strength in the *DirectLiNGAM* algorithm was determined by applying ordinary least squares regression to compute the edge weights, thereby quantifying the causal effect strength.

In the CMM network, the in-degree (Equation 2), out-degree (Equation 3), degree centrality (Equations 4 and 5), betweenness centrality (Equations 6 and 7), and closeness centrality (Equations 8 and 9) were computed as the metric values for each node. Finally, feature importance ranking was performed, and the top eight feature variables were selected.


$$\begin{array}{c} x_{-j}=a\cdot x_{j}+r^{(j)} \end{array}$$ (1)

Herein, a represents the regression coefficient vector, and *r* ^*(j)*^ denotes the residual vector. The algorithm identifies the root node by testing the statistical independence between *x*_*j*_ and *r* ^*(j)*^.


$$\begin{array}{c} ID(v)=\sum_{u\in V}A_{uv} \end{array}$$ (2)

$$\begin{array}{c} OD(v)=\sum_{u\in V}A_{vu} \end{array}$$ (3)

A directed graph is represented as G=(V,E), where *V* denotes the set of nodes (corresponding to variables in this paper) and *E* denotes the set of directed edges (representing structural relationships between variables in this paper). Let n=|V| be the total number of nodes. *A* is the adjacency matrix, such that if there exists an edge from node *u* to node *v*, then $A_{uv}=\text{1}$, In a weighted network, $A_{uv}$ represents the weight of the edge.


$$\begin{array}{c} C_{D}(v)=ID(v)+OD(v)=\sum_{u\in V}(A_{uv}+A_{vu})\;\; \end{array}$$ (4)

$$\begin{array}{c} C\prime_{D}(v)=\frac{C_{D}(v)}{2(n-1)}\;\; \end{array}$$ (5)

The maximum number of connections in the network is 2(n−1), and $C\prime_{D}(v)$ is the normalization of $C_{D}(v)$.


$$\begin{array}{c} C_{B}(v)=\sum_{s\text{≠}v\text{≠}t\in V}\frac{\sigma_{st}(v)}{\sigma_{st}} \end{array}$$ (6)

$$\begin{array}{c} C_{B}^{\prime }(v)=\frac{C_{B}(v)}{(n-1)(n-2)}\;\; \end{array}$$ (7)

The total number of shortest paths from node *s* to node *t* is denoted as $\sigma_{st}$, and the number of shortest paths from node *s* to node *t* that pass through node *v* is represen*t*ed as $\sigma_{st}(v)$. $C\prime_{B}(v)$ is the normalized version of $C_{B}(v)$.


$$\begin{array}{c} C_{C}(v)=\frac{1}{\sum_{u\in V,u\text{≠}v}d(v,u)} \end{array}$$ (8)

$$\begin{array}{c} C^{\prime }(v)=\frac{n-1}{\sum_{u\in V,u\text{≠}v}d(v,u)} \end{array}$$ (9)

The shortest path distance from node *v* to node *u* is denoted as *d* (*v*,*u*). $C\prime_{C}(v)$ represents the normalized version of $C_{C}(v)$.

### 2.4. Construction of the digital marker and the CMM subgroup stratification

This section consists of two sequential steps. In the first step, ten machine learning algorithms (*Random Forest*, *XGBoost*, *MLP*, *KNN*, *Gradient Boosting*, *SVC*, *Linear Regression*, *Ridge*, *Elastic Net*, and *Lasso*) are applied, using the top seven clinical laboratory parameters and demographic characteristics ranked by network importance as input variables to predict the probability of death for each individual, thereby generating a continuous digital marker to quantify mortality risk. In the second step, based on tertiles of this digital marker, the population is stratified into three ordered subgroups (low, middle, and high), forming a three-class classification task.

Participants were first stratified by their year of enrolment and follow-up. Within each year stratum, a 7:3 random split was performed independently to assign participants to the global training set (Outer Train) and the global test set (Outer Test). The test set was completely excluded from any data preprocessing (including missing value imputation and standardisation), feature selection, causal network construction, and hyperparameter optimisation. It was used solely for final digital marker generation and model performance evaluation, thereby strictly simulating a real-world prospective clinical prediction scenario.

Feature engineering involved selecting specific laboratory variables and incorporating all demographic variables. The preprocessing pipeline directly passed numerical variables while applying one-hot encoding to categorical variables. Model training employed a nested 5‑fold cross‑validation framework: the outer 5‑fold loop assessed generalisation performance, while the inner 5‑fold loop performed grid search for hyperparameters. Within each fold, missing value imputation (median), standardisation, and feature selection were independently performed on the training sub‑set and applied to the corresponding validation sub‑set, completely preventing cross‑fold information leakage. For tree-based models, *Random Forest* and *XGBoost* were configured with 100 base learners (*n_estimators* = 100) and a fixed random seed (*random_state* = 50) to ensure reproducibility. For linear models, the regularization strengths of *Ridge*, *Lasso* and *Elastic Net* were set to 1.0, 0.1 and 0.1, respectively, which were determined by grid search with cross-validation on the training set. *SVC* with a radial basis function kernel was utilized, *KNN* employed 5 nearest neighbors(selected via cross-validation), and the *MLP* was configured with a two-layer hidden structure comprising 100 and 50 neurons, respectively. The digital marker was defined as the disease count predicted by the optimal machine learning model, serving as a continuous measure of systemic multimorbidity burden. Based on the distribution of this marker, patients were stratified into three ordered subgroups: low (≤*P*_*33*_), middle (between *P*_*33*_ to *P*_*67*_), and high (>*P*_*67*_). This staging integrates multiple clinical and demographic characteristics as a composite indicator to enhance clinical interpretability and support subgroup‑based intervention. Model performance was evaluated using *Bootstrap* resampling (1,000 iterations) to compute 95% confidence intervals for each metric, including *AUC* (*C‑index*), *PR‑AUC*, *Brier Score* (Equation 10), *Log Loss* (Equation 11).


$$\begin{array}{c} Brier\;score=\frac{\text{1}}{N}\sum_{i=\text{1}}^{N}{\left(p_{i}-y_{i}\right)}^{\text{2}} \end{array}$$ (10)

$$\begin{array}{c} LogLoss=-\frac{\text{1}}{N}\sum_{i=\text{1}}^{N}\left[y_{i}\;log\left(p_{i}\right)+\left(\text{1}-y_{i}\right)log\left(\text{1}-p_{i}\right)\right] \end{array}$$ (11)

### 2.5. Statistical methods

Descriptive statistics were conducted to estimate the *Mean (Standard Deviation*) for continuous variables. *Frequency* (*Proportion*) was calculated for categorical variables. To determine differences in the distribution of digital marker scores with respect to characteristics, *student t* test and *Pearson* test were utilized. The association between demographic characteristics, clinical laboratory parameters, diseases status and the digital marker was determined using *Multiple Linear Regression* with robust standard errors. All tests were based on two-sided tests with a 95% confidence interval. Missing data were handled by complete-case analysis, as the proportion of missing values was below 5% for all variables and the missingness was determined to be random based on Little’s MCAR test. To evaluate the robustness of the estimated network structure, we performed bootstrap stability analysis with 1,000 resamples (85% of the original sample size). For each bootstrap sample, the DirectLiNGAM algorithm was re-estimated, and edge occurrence frequencies and node centrality rankings were recorded. A directed edge was considered stable if it appeared in ≥85% of the bootstrap replications, and node centrality consistency was assessed using Kendall’s τ coefficient. The linearity assumption was examined by inspecting residual plots and calculating variance inflation factors, while the non-Gaussianity of residuals was verified by Shapiro-Wilk tests all continuous variables) for all continuous variables, confirming the suitability of DirectLiNGAM for the present dataset.

## 3. Results

### 3.1. Demographic characteristics

A total of 8,226 participants were included in this analysis, with 4352 females (52.91%) and 3874 males (47.09%). The mean age of the participants was 60.547 (9.861). Among all participants, 62.12% were from rural areas, 54.72% had a formal schooling, and 13.19% were unmarried. The detailed information is presented in Table 2.

### 3.2. CMM network constructed and network traits

**Fig 2:**
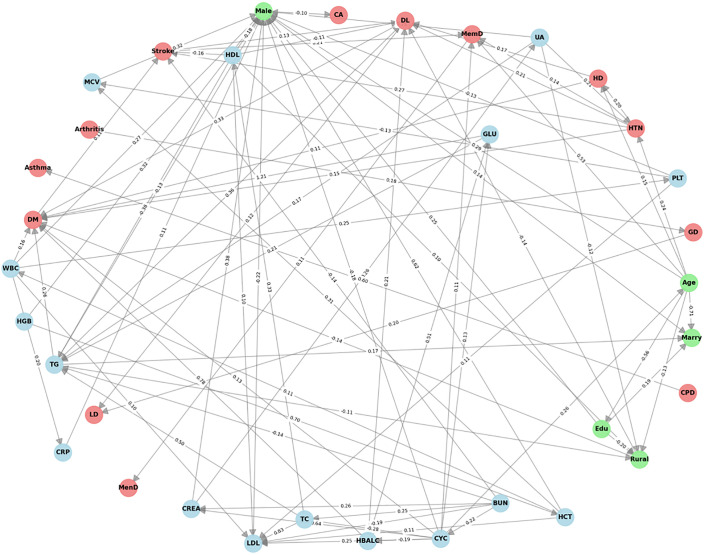
CMM network constructed with *DirectLiNGAM.*

The CMM network constructed based on the *DirectLiNGAM* algorithm is detailed in Fig 2. The characteristics of the network are presented in S1 Table. To assess the stability of the estimated network structure, we performed bootstrap analysis with 1,000 resamples among the train group. The core directed edges appeared in over 85% of the bootstrap replications, indicating substantial stability. Node centrality rankings also demonstrated high consistency, with Kendall’s τ coefficients of 0.662(0.077), for degree centrality, 0.461(0.105) for betweenness centrality, and 0.599(0.092) for closeness centrality between the original and bootstrap-averaged rankings. These results suggest that the key structural associations identified in the CMM network are robust to sampling variability.

**Fig 3:**
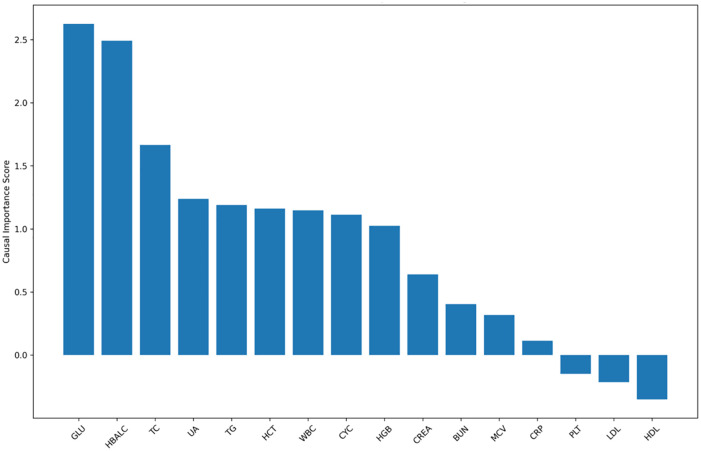
Lab variables causal importance ranking.

As can be seen from Fig 2. and Fig 3. the top three disease nodes in terms of in-degree are Diabetes Mellitus (DM), Memory Disorders (MemD), and Dyslipidemia (DL), which serve as primary outcomes for other nodes in the CMM network. The top five nodes in terms of out-degree are Total Cholesterol (TC), Glycated Hemoglobin A1c (HBALC), Fasting Blood Glucose (GLU), Hematocrit (HCT), and Hemoglobin (HGB), which act as the main initiating factors in the CMM network. The top five nodes in terms of degree centrality are Male (gender), Triglycerides (TG), Diabetes Mellitus (DM), Cystatin C (CYC), and DL, which serve as core hubs in the evolution of the CMM network. The top five nodes in terms of betweenness centrality are Male, Stroke, TG, DL, and DM, which lie on critical paths in the evolution of the CMM network. The top five nodes in terms of closeness centrality are Male, DM, Married (marital status), Stroke, and Cancer (CA), which have the shortest average distances to other nodes and are the primary influencers of the evolutionary rate of the CMM network.

### 3.3. Feature importance ranking and selection of key feature variables

The results of feature importance ranking are detailed in Fig 3. In the evolution of the CMM network, the top seven clinical laboratory parameters ranked by their importance from high to low are as follows: GLU, HBALC, TC, serum uric acid（UA）, TG, Hematocrit (HCT), White Blood Cells (WBC).

### 3.4. Model performance evaluation for the digital marker

Table 1 presents the predictive performance of machine learning models for death as the outcome variable on the independent test set. As shown in Table 1, the *Ridge* model achieved the best performance, with an *AUC* of 0.8515 (95% *CI*: 0.8244, 0.8750), *PR‑AUC* of 0.5302 (95% *CI*: 0.4697, 0.5928), *Brier Score* of 0.0780 (95% *CI*: 0.0709, 0.0850), and *Log Loss* of 0.2676 (95% *CI*: 0.2453, 0.2908), indicating good discriminative ability. The *pseudo R²* was 0.2763(0.2240, 0.3171). S1 Fig presents the calibration curves of selected machine learning models. Overall, the *Linear Regression*, *Ridge*, *Elastic Net*, etc, were well-calibrated, whereas *KNN* and *MLP* showed slight deviations in the high-risk range. S2 Table presents the predictive performance of the five baseline models on the independent test set. The mean digital markers scores for CMM in the Low group, Middle group, and High group are 0.016 (0.008), 0.058 (0.019), and 0.296 (0.197), respectively.The actual mortality rate in the test set was 0.122, which was closely approximated by the mean predicted probability (0.124) of the digital marker constructed in this study, indicating good model calibration and a certain degree of clinical net benefit (see S2 Fig).

**Table 1:** Predictive performance of machine learning models on the independent test set.

Models	*AUC (95% CI*)	*PR-AUC (95% CI*)	*Brier Score (95% CI*)	*Log Loss (95% CI*)	*Pseudo R² (95% CI*)
***Random Forest***	0.8298 (0.7998, 0.8537)	0.4724 (0.4165, 0.5360)	0.0857 (0.0774, 0.0933)	0.2925 (0.2703, 0.3128)	0.2090 (0.1782, 0.2346)
***XGBoost***	0.8422 (0.8169, 0.8647)	0.4936 (0.4323, 0.5574)	0.0808 (0.0734, 0.0881)	0.2744 (0.2515, 0.2956)	0.2580 (0.2132, 0.3003)
***MLP***	0.7508 (0.7193, 0.7804)	0.3812 (0.3201, 0.4486)	0.1167 (0.1057, 0.1289)	0.6118 (0.5387, 0.6959)	0 (0, 0)
***KNN***	0.7274 (0.6947, 0.7583)	0.2994 (0.2494, 0.3590)	0.0981 (0.0890, 0.1065)	1.3845 (1.1227, 1.6113)	0 (0, 0)
***Gradient Boosting***	0.8415 (0.8139, 0.8648)	0.4770 (0.4143, 0.5377)	0.0817 (0.0738, 0.0891)	0.2773 (0.2548, 0.2998)	0.2502 (0.2026, 0.2933)
***SVC***	0.7994 (0.7672, 0.8269)	0.4690 (0.4018, 0.5300)	0.0846 (0.0764, 0.0924)	0.2942 (0.2707, 0.3166)	0.2045 (0.1588, 0.2434)
***Linear Regression***	0.8514 (0.8247, 0.8748)	0.5299 (0.4695, 0.5929)	0.0780 (0.0708, 0.0850)	0.2676 (0.2451, 0.2907)	0.2763 (0.2230, 0.3179)
***Ridge***	0.8515 (0.8244, 0.8750)	0.5302 (0.4697, 0.5928)	0.0780 (0.0709, 0.0850)	0.2676 (0.2453, 0.2908)	0.2763 (0.2240, 0.3171)
***ElasticNet***	0.8515 (0.8243, 0.8749)	0.5292 (0.4684, 0.5921)	0.0780 (0.0709, 0.0849)	0.2677 (0.2455, 0.2903)	0.2761 (0.2252, 0.3161)
***Lasso***	0.8509 (0.8240, 0.8742)	0.5252 (0.4640, 0.5878)	0.0784 (0.0712, 0.0852)	0.2686 (0.2466, 0.2903)	0.2738 (0.2245, 0.3120)

### 3.5. Characteristics of the subjects in different digital markers scores

The characteristics of subjects at different CMM subgroups are detailed in Table 2. As shown in Table 2, the results of multiple comparisons indicate that there are significant differences in digital markers scores among different genders, marital statuses, residential regions, and educational levels (*P* < 0.001). The digital marker scores are significantly correlated with age, as well as with WBC, GLU, TG, and HBALC (*P* < 0.001). Additionally, the digital marker scores demonstrates significant differences across most chronic diseases included in this paper (*P* < 0.001).

**Table 2:** Characteristics of the subjects in different CMM disease subgroups.

	*Digital marker score*		
	*Mean (SD*)	*t/r*	*P*
**Gender**		15.444	<0.001
**Females**	0.096 (0.143)		
**Males**	0.154 (0.189)		
**Marital status**		20.171	<0.001
**Unmarried**	0.248 (0.227)		
**Married**	0.105 (0.149)		
**Education level**		25.533	<0.001
**Without formal schooling**	0.176 (0.200)		
**With formal schooling**	0.080 (0.121)		
**Living area**		7.458	<0.001
**Urban**	0.106 (0.151)		
**Rural**	0.134 (0.178)		
**Age**		0.654	<0.001
**WBC**		0.131	<0.001
**MCV**		0.074	<0.001
**PLT**		−0.002	0.824
**BUN**		0.178	<0.001
**GLU**		0.213	<0.001
**CREA**		0.219	<0.001
**TC**		0.013	0.221
**TG**		−0.063	<0.001
**HDL**		0.044	<0.001
**LDL**		0.031	0.005
**CRP**		0.272	<0.001
**HBALC**		−0.049	<0.001
**UA**		0.055	<0.001
**HCT**		−0.065	<0.001
**HGB**		−0.008	0.465
**CYC**		0.614	<0.001

The results of the *multiple linear regression* analysis are detailed in Table 3. As shown in Table 3, after adjusting for confounding factors, among the clinical laboratory parameters, GLU (*β* = 0.284), TG (*β* = -0.085), HBALC (*β* = -0.172), UA (*β* = -0.090), HCT (*β* = -0.084), HGB (*β* = 0.033), and CYC (*β* = 0.492) all exhibited significant associations with the outcome (*P* < 0.001).

**Table 3:** The association between the characteristics and the digital marker scores.

	*β* ^*!*^	*P*	*95% CI*	*VIF*
**Contents**	–	<0.001	−0.445, −0.384	–
**Male**	0.204	<0.001	0.064, 0.073	1.693
**Marry**	−0.093	<0.001	−0.052, −0.041	1.131
**Rural**	0.058	<0.001	0.016, 0.024	1.102
**Edu**	−0.113	<0.001	−0.042, −0.034	1.264
**Age**	0.37	<0.001	0.006, 0.007	1.55
**WBC**	0.031	<0.001	0.002, 0.004	1.228
**MCV**	0.027	<0.001	>0.001, 0.001	1.198
**PLT**	0.066	<0.001	>0.001, > 0.001	1.176
**BUN**	−0.056	<0.001	−0.002, −0.002	1.316
**GLU**	0.284	<0.001	0.001, 0.002	1.723
**CREA**	−0.162	<0.001	−0.096, −0.080	2.112
**TC**	0.17	<0.001	0.001, 0.001	16.413
**TG**	−0.085	<0.001	>−0.001, >−0.001	5.377
**HDL**	0.004	0.626	>−0.001, < 0.001	3.038
**LDL**	−0.171	<0.001	−0.001, −0.001	12.62
**CRP**	0.139	<0.001	0.003, 0.004	1.113
**HBALC**	−0.172	<0.001	−0.032, −0.027	1.831
**UA**	−0.09	<0.001	−0.013, −0.010	1.401
**HCT**	−0.084	<0.001	−0.003, −0.002	2.632
**HGB**	0.033	<0.001	0.001, 0.004	2.556
**CYC**	0.492	<0.001	0.272, 0.289	2.307
**HTN**	0.039	<0.001	0.010, 0.018	1.187
**DM**	0.02	0.001	0.005, 0.020	1.367
**CA**	0.062	<0.001	0.078, 0.109	1.011
**CPD**	0.051	<0.001	0.020, 0.032	1.238
**HD**	0.044	<0.001	0.016, 0.026	1.159
**Stroke**	0.078	<0.001	0.061, 0.080	1.054
**MenD**	0.02	<0.001	0.013, 0.040	1.017
**Arthritis**	−0.049	<0.001	−0.020, −0.013	1.079
**DL**	−0.046	<0.001	−0.027, −0.017	1.208
**LD**	0.01	0.05	−0.000, 0.016	1.041
**CKD**	−0.003	0.593	−0.008, 0.005	1.076
**GD**	−0.022	<0.001	−0.012, −0.004	1.087
**Asthma**	0.076	<0.001	0.049, 0.066	1.207
**MemD**	0.083	<0.001	0.082, 0.105	1.048

^**!**^*R*^*2*^=0.780，*F*=831.810，*P<*0.001.

## 4. Discussion

This study identifies that abnormalities in clinical laboratory parameters, specifically TC, HBALC, GLU, HCT, and HGB, serve as the core driving factors for the evolution of the CMM network. These parameters might trigger subsequent pathophysiological processes and rank highest in Outdegree within the network. Additionally, DM, MemD, and DL occupy leading positions in Indegree, indicating they might are common endpoints. Centrality measures further highlight Male, TG, DM, CYC, DL, Stroke, Married, and CA might be widely connected or bridging nodes. Notably, the key clinical laboratory parameters (GLU, HBALC, TC, UA, TG, HCT, and WBC) identified by network analysis are highly consistent with those from traditional linear regression, and *Ridge regression* outperforms other machine learning models in predicting death. Based on the digital marker from *Ridge regression*, adjusted linear models show significant differences across genders, marital statuses, regions, and age groups.

Abnormalities in TC, TG, HBALC, and GLU suggest glycolipid metabolic disorders, which aligns with prior evidence that the triglyceride-glucose index predicts the risk of developing comorbid combinations including cardiovascular diseases, diabetes mellitus, and chronic kidney diseases [30–33], and that the hemoglobin glycation index predicts all-cause mortality in patients with diabetes mellitus complicated by cardiovascular diseases [34]. These abnormalities are linked to insulin resistance, systemic inflammation, and oxidative stress, which are shared mechanisms among various diseases within the CMM framework [35,36]. Abnormalities in HCT and HGB indicate anemia, hemodynamic changes, and chronic hypoxia, consistent with studies showing that both HCT, HGB, and their ratio predict adverse outcomes in metabolic or cardiovascular diseases [37–39]. They are associated with blood hyperviscosity, insulin resistance, oxidative stress, and endothelial function dysfunction, which are core shared mechanisms in CMM [40]. Abnormalities in UA suggest purine metabolic disorders and impaired glomerular filtration, similar to findings that the uric acid/creatinine ratio [41,42] and the estimated glomerular filtration rate based on cystatin C [43, 44], predict adverse outcomes in cardiovascular diseases. UA and WBC are linked to systemic inflammation, uremic toxins accumulation, oxidative stress, and endothelial dysfunction, which are key drivers within the CMM framework [45].

Cross-validation consistently identified GLU, HBALC, TC, UA, HCT, TG, HGB, WBC, and CYC in both network and linear regression models, validating their robust intrinsic connections within the CMM disease network. These indicators reflect metabolic disorders, oxidative stress, chronic inflammation, and abnormal blood rheology. The digital marker generated based on these clinical laboratory parameters and the CMM subgroups derived from it exhibit a certain degree of reliability. The digital marker scores demonstrate significant distribution differences across populations with varying genders, marital statuses, and residential regions. This paper found that males have lower digital marker scores compared to females, while older individuals have higher scores than younger ones. Currently, there is a lack of analysis on the differences in CMM network characteristics among different populations, so this paper only conducts a comparative analysis with existing relevant literature. Previous research has found that males and females exhibit certain differences in various cardiovascular and metabolic diseases, which is similar to the gender differences observed in this paper [46]. Additionally, the finding that older individuals have higher‑scoring CMM subgroups (i.e., higher digital marker scores) in this paper aligns with previous research results indicating higher incidences of cardiovascular and metabolic diseases, as well as CMM, in older age groups [47,48].

In clinical practice, TC, HBALC, GLU, HCT, and HGB may be considered as the primary clinical laboratory parameters for risk early warning and early intervention. Diseases such as DM, metabolic MemD, and DL, which are common endpoints in the CMM network structure, may serve as core targets for comprehensive clinical treatment and long-term management. Factors including gender (Male), TG, DM, CYC, and age are widely connected to numerous nodes within the CMM network, and changes in their states can rapidly affect the overall network stability, thus holding significant reference value in assessing the systemic state of CMM. Factors such as TG and WBC regulate the efficiency of influence between different nodes. Interventions targeting these key pathway nodes may effectively block specific pathological processes and prevent the occurrence of adverse clinical outcomes. In contrast, diseases like DM, asthma, and CA are associated with the overall connectivity of the CMM network and may serve as critical disease processes requiring focused control in prevention and treatment efforts.

Although the three subgroups were derived from the predicted disease count, the digital marker integrates multidimensional clinical laboratory and demographic information, thereby capturing systemic pathophysiological states beyond simple disease enumeration. To further demonstrate its clinical utility beyond disease count stratification, future studies can evaluate its association with independent clinical endpoints such as hospitalization, cardiovascular events, and mortality. Such validation will establish the subgroup definition as a clinically meaningful indicator rather than a convenient statistical binning. The analysis of CMM network subgroups in this paper has the following limitations. First, constrained by the cross-sectional paper design employed, while this paper can delineate the network structure of CMM at a specific time point, it struggles to capture its dynamic evolutionary trajectory over time or rigorously infer causal directions between nodes. Consequently, the classification of network subgroups relies more on topological inference than direct observation. Second, the data utilized in this research are limited in breadth, length, and depth. Specifically, the relatively homogeneous sample source and limited population coverage may compromise the model’s generalizability. Second, the application of DirectLiNGAM relies on several assumptions, including linearity, non-Gaussian noise, acyclicity, and the absence of unmeasured confounders. While our data satisfied the non-Gaussian condition and the linearity assumption was supported by model diagnostics, the possibility of undetected nonlinear relationships remains. Moreover, the acyclicity assumption, although reasonable for cross-sectional data, may not fully capture potential feedback loops that could exist over longer time courses. The presence of unmeasured confounders, such as genetic predisposition or detailed lifestyle factors, cannot be entirely excluded. Therefore, the directed edges in this network should be interpreted as structural associations rather than definitive causal mechanisms. Moreover, the absence of systematic long-term follow-up data hinders the validation of temporal patterns in network change over extended periods. Additionally, the predominance of conventional clinical laboratory parameters in the dataset, without incorporating more molecular biomarkers reflecting specific pathophysiological processes, restricts in-depth exploration of the underlying driving mechanisms of the network. At the analytical level, although this paper identifies key nodes and community structures within the network, it has yet to effectively reveal the temporal patterns of different functional communities and their dynamic interaction modes. This limitation leaves our understanding of the evolutionary pathway of CMM from local dysregulation to systemic disorder still constrained. In the future, through the establishment of multicenter, prospective long-term follow-up cohorts and the integration of multi-omics data, combined with the application of nested cross-validation or external validation, it is expected that the complete dynamic landscape of the CMM network can be revealed comprehensively on a more solid evidentiary basis, while further supporting the generalizability of the research findings.

## 5. Conclusions

Based on the analysis combining directed cyclic networks with machine learning, this paper reveals the complex topological associations and interactive relationships among demographic characteristics, clinical laboratory parameters, and disease status features within the CMM network. The findings indicate that total cholesterol, glycated hemoglobin, fasting blood glucose, hematocrit, and hemoglobin may play pivotal driving roles in the network structure of CMM, while factors such as male gender, triglycerides, diabetes mellitus, cystatin C, and age serve as bridges and hubs within the network. GLU, HBALC, TC, UA, HCT, TG, HGB, WBC, and CYC are identified as key clinical laboratory parameters in characterizing CMM subgroups, describing the cross‑sectional status of the CMM network from multiple perspectives, including disorders of glucose and lipid metabolism, abnormal blood rheology, renal impairment, and chronic inflammatory states. By integrating key clinical laboratory parameters and demographic characteristics, the digital marker provides a continuous and multidimensional characterization of the systemic state of chronic disease multimorbidity. The three subgroups delineated based on this marker may serve as a practical tool for depicting cross‑sectional disease status, with potential applications in early identification and subgroup-based intervention. In comparison to classification based solely on disease count, this marker demonstrates certain advantages in integrating multidimensional information. This paper provides a reliable indicator for the clinical characterization of CMM network subgroups and offers a referable paradigm for the holistic treatment and intervention of CMM. Future research should focus on constructing cross-regional, long-term clinical follow-up cohorts and integrating multi-omics data to jointly uncover the evolutionary patterns of the CMM network at both global and local scales.

## Supporting information

S1 Table Described the characteristics of the CMM network. (DOCX)

S2 Table Presents the predictive performance of the five baseline models on the independent test set. The results showed a clear gradient of improvement. M1 (simple disease count) had the weakest discrimination (*AUC* = 0.589, *PR-AUC* = 0.147) and poor calibration (*Brier Score* = 0.125, *Log Loss* = 1.01). M2 (age + sex) and M3 (age + sex + basic biochemistry) sequentially improved, with *AUC* increasing to 0.797 and 0.824, respectively, and *PR-AUC* reaching 0.395 and 0.422, while calibration errors decreased markedly. M4 (full-variable logistic regression) and M5 (the best machine learning model *Ridge*) performed almost identically, achieving the best discrimination (*AUC* = 0.8515, *PR-AUC* = 0.530) and the smallest calibration errors (*Brier Score* = 0.078, *Log Loss* = 0.268). The calibration intercepts were positive for all models (0.15–0.39), indicating a slight overall overestimation of mortality risk. *Pseudo R²* increased from 0.00 in M1 to 0.276 in M4/M5, suggesting that the latter explained about 27.6% of the variability in death risk. In summary, the full-variable logistic regression and the best machine learning model had no significant difference in predictive performance, and both substantially outperformed the simplified models based only on demographic or basic biochemical variables.
(DOCX)

S1 Fig Shows the calibration curves of selected machine learning models. (TIF)

S2 Fig Shows the decision curve analysis for identifying high-risk patients, illustrating the net clinical benefit of the digital marker (best model) and the four baseline models across different threshold probabilities. (TIF)

S3 Fig Shows the association between the characteristics and the digital marker scores. (TIF)
