# Supplementary material for: A digital marker for stratifying cardiovascular metabolic comorbidities among the middle-aged and elderly adults
Source: PLOS Digit Health. 2026 Jul 2;5(7):e0001528. doi: 10.1371/journal.pdig.0001528 (PMC13327254; doi:10.1371/journal.pdig.0001528)
Supplement: S1 Table — (DOCX) [file pdig.0001528.s001.docx]

**S1 Tabel Characteristics of the CMM network**

|  | **In degree** | **Out degree** | **Degree centrality** | **Betweenness centrality** | **Closeness centrality** |
| --- | --- | --- | --- | --- | --- |
| **WBC** | 0.1060 | 0.9794 | 0.1818 | 0.0114 | 0.0404 |
| **MCV** | 0.1824 | 0.3177 | 0.0909 | 0 | 0.0808 |
| **PLT** | 0.2513 | -0.1500 | 0.1212 | 0.0009 | 0.0455 |
| **BUN** | 0 | 0.2648 | 0.1818 | 0 | 0 |
| **GLU** | 0.6354 | 1.4131 | 0.1212 | 0.0052 | 0.0682 |
| **CREA** | 0.8984 | 0.6363 | 0.1212 | 0.0028 | 0.0606 |
| **TC** | -0.0234 | 1.6634 | 0.1515 | 0.0024 | 0.0606 |
| **TG** | 0.3597 | 0.5508 | 0.3030 | 0.0213 | 0.1763 |
| **HDL** | 0.3314 | -0.6287 | 0.2121 | 0.0049 | 0.0545 |
| **LDL** | 1.3231 | -0.2152 | 0.2424 | 0.0011 | 0.2155 |
| **CRP** | 0.1961 | 0.1131 | 0.0606 | 0 | 0.0455 |
| **HBALC** | -0.1903 | 1.5001 | 0.1212 | 0.0019 | 0.0404 |
| **UA** | 0.2581 | 1.0170 | 0.1515 | 0.0099 | 0.0545 |
| **HCT** | 0.7031 | 1.1524 | 0.1515 | 0.0085 | 0.0303 |
| **HGB** | 0 | 1.0242 | 0.0606 | 0 | 0 |
| **CYC** | 0.2245 | 0.8689 | 0.3030 | 0.0071 | 0.0303 |
| **HTN** | 0.4555 | 0.9597 | 0.2121 | 0.0114 | 0.0842 |
| **DM** | 2.9946 | 0.1101 | 0.3030 | 0.0194 | 0.3386 |
| **CA** | -0.1034 | 0 | 0.0303 | 0 | 0.2605 |
| **CPD** | 0 | 0.6012 | 0.0303 | 0 | 0 |
| **HD** | 0.3421 | 0.2823 | 0.1212 | 0.0009 | 0.0909 |
| **Stroke** | 0.2096 | 0.2100 | 0.1818 | 0.0256 | 0.2879 |
| **MenD** | 0.1060 | 0 | 0.0303 | 0 | 0.1856 |
| **Arthritis** | 0 | 0.1760 | 0.0303 | 0 | 0 |
| **DL** | 0.6941 | 0.5907 | 0.2727 | 0.0207 | 0.2582 |
| **LD** | 0.3212 | 0 | 0.0606 | 0 | 0.2136 |
| **CKD** | 0 | 0 | 0 | 0 | 0 |
| **GD** | 0.1760 | 0.1968 | 0.0606 | 0.0009 | 0.0303 |
| **Asthma** | 0.6012 | 0 | 0.0303 | 0 | 0.0303 |
| **MemD** | 0.9833 | 0.1060 | 0.1515 | 0.0189 | 0.2377 |
| **Age** | 0.2648 | -0.1925 | 0.2424 | 0.0066 | 0.0404 |
| **Male** | 2.4735 | 0.0393 | 0.4848 | 0.0260 | 0.4269 |
| **Marry** | -0.2079 | 0 | 0.1212 | 0 | 0.3126 |
| **Rural** | -0.4500 | -0.2756 | 0.2121 | 0.0088 | 0.2037 |
| **Edu** | -0.5649 | 0.2399 | 0.1212 | 0 | 0.0455 |
