# Supplementary material for: A digital marker for stratifying cardiovascular metabolic comorbidities among the middle-aged and elderly adults
Source: PLOS Digit Health. 2026 Jul 2;5(7):e0001528. doi: 10.1371/journal.pdig.0001528 (PMC13327254; doi:10.1371/journal.pdig.0001528)
Supplement: S2 Table — The results showed a clear gradient of improvement. M1 (simple disease count) had the weakest discrimination (AUC = 0.589, PR-AUC = 0.147) and poor calibration (Brier Score = 0.125, Log Loss = 1.01). M2 (age + sex) and M3 (age + sex + basic biochemistry) sequentially improved, with AUC increasing to 0.797 and 0.824, respectively, and PR-AUC reaching 0.395 and 0.422, while calibration errors decreased markedly. M4 (full-variable logistic regression) and M5 (the best machine learning model Ridge) performed almost identically, achieving the best discrimination (AUC = 0.8515, PR-AUC = 0.530) and the smallest calibration errors (Brier Score = 0.078, Log Loss = 0.268). The calibration intercepts were positive for all models (0.15–0.39), indicating a slight overall overestimation of mortality risk. Pseudo R² increased from 0.00 in M1 to 0.276 in M4/M5, suggesting that the latter explained about 27.6% of the variability in death risk. In summary, the full-variable logistic regression and the best machine learning model had no significant difference in predictive performance, and both substantially outperformed the simplified models based only on demographic or basic biochemical variables. (DOCX) [file pdig.0001528.s002.docx]

**S2 Table Predictive performance of five baseline models on the independent test set**

| **Models** ^ | ***AUC(***95% CI) | ***PR-AUC(***95% CI) | ***Brier Score(***95% CI) | ***Log Loss(***95% CI) | ***Pseudo R²(***95% CI) |
| --- | --- | --- | --- | --- | --- |
| **M1_Count** | 0.5892(0.5582-0.6213) | 0.1473(0.1290-0.1693) | 0.1253(0.1157-0.1344) | 1.0126(0.8133-1.1911) | 0.0000(0.0000-0.0000) |
| **M2_AgeGender** | 0.7967(0.7681-0.8233) | 0.3947(0.3368-0.4505) | 0.0893(0.0808-0.0968) | 0.3033(0.2803-0.3247) | 0.1798(0.1467-0.2096) |
| **M3_BasicBio** | 0.8238(0.7963-0.8481) | 0.4221(0.3591-0.4816) | 0.0867(0.0784-0.0950) | 0.2920(0.2683-0.3134) | 0.2105(0.1726-0.2418) |
| **M4_FullLogistic** | 0.8514(0.8247-0.8748) | 0.5299(0.4695-0.5929) | 0.0780(0.0708-0.0850) | 0.2676(0.2451-0.2907) | 0.2763(0.2230-0.3179) |
| **M5_ML_Best** | 0.8515(0.8244-0.8750) | 0.5302(0.4697-0.5928) | 0.0780(0.0709-0.0850) | 0.2676(0.2453-0.2908) | 0.2763(0.2240-0.3171) |

^ M1, raw disease count ; M2, age and sex only; M3, age, sex, fasting glucose (GLU) and glycated hemoglobin (HbA1c); M4, full‑variable conventional logistic regression (all laboratory and demographic variables); M5, the machine learning digital marker of the present study (Ridge regression).
