# Supplementary figures and images for: A digital marker for stratifying cardiovascular metabolic comorbidities among the middle-aged and elderly adults
Source: PLOS Digit Health. 2026 Jul 2;5(7):e0001528. doi: 10.1371/journal.pdig.0001528 (PMC13327254; doi:10.1371/journal.pdig.0001528)

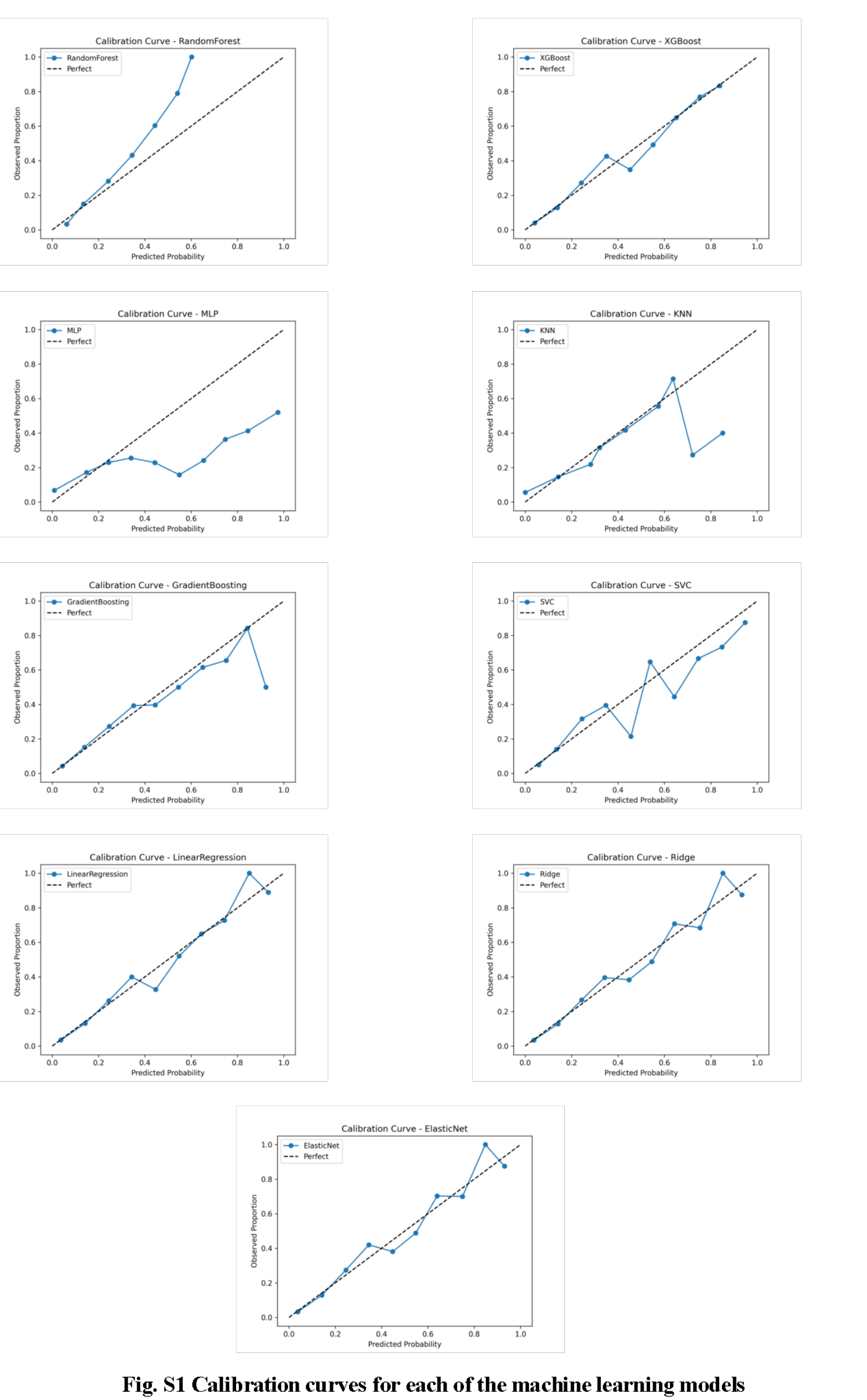

Supplement: S1 Fig — (TIF) [file pdig.0001528.s003.tif]

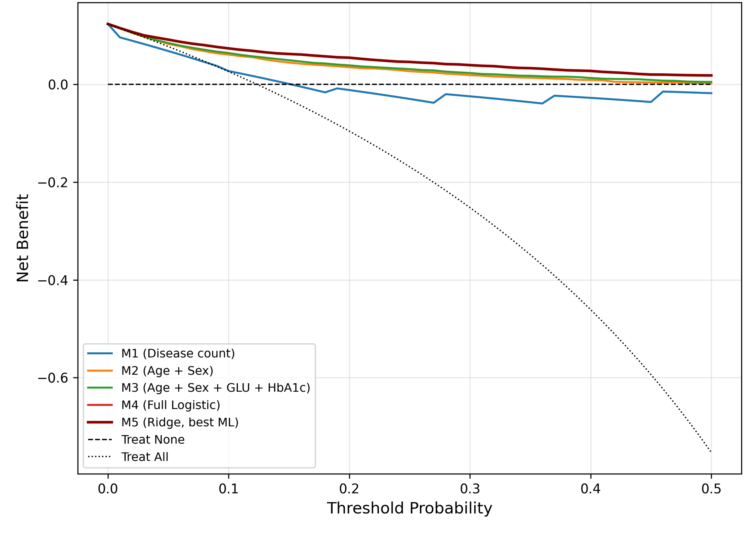

Supplement: S2 Fig — (TIF) [file pdig.0001528.s004.tif]

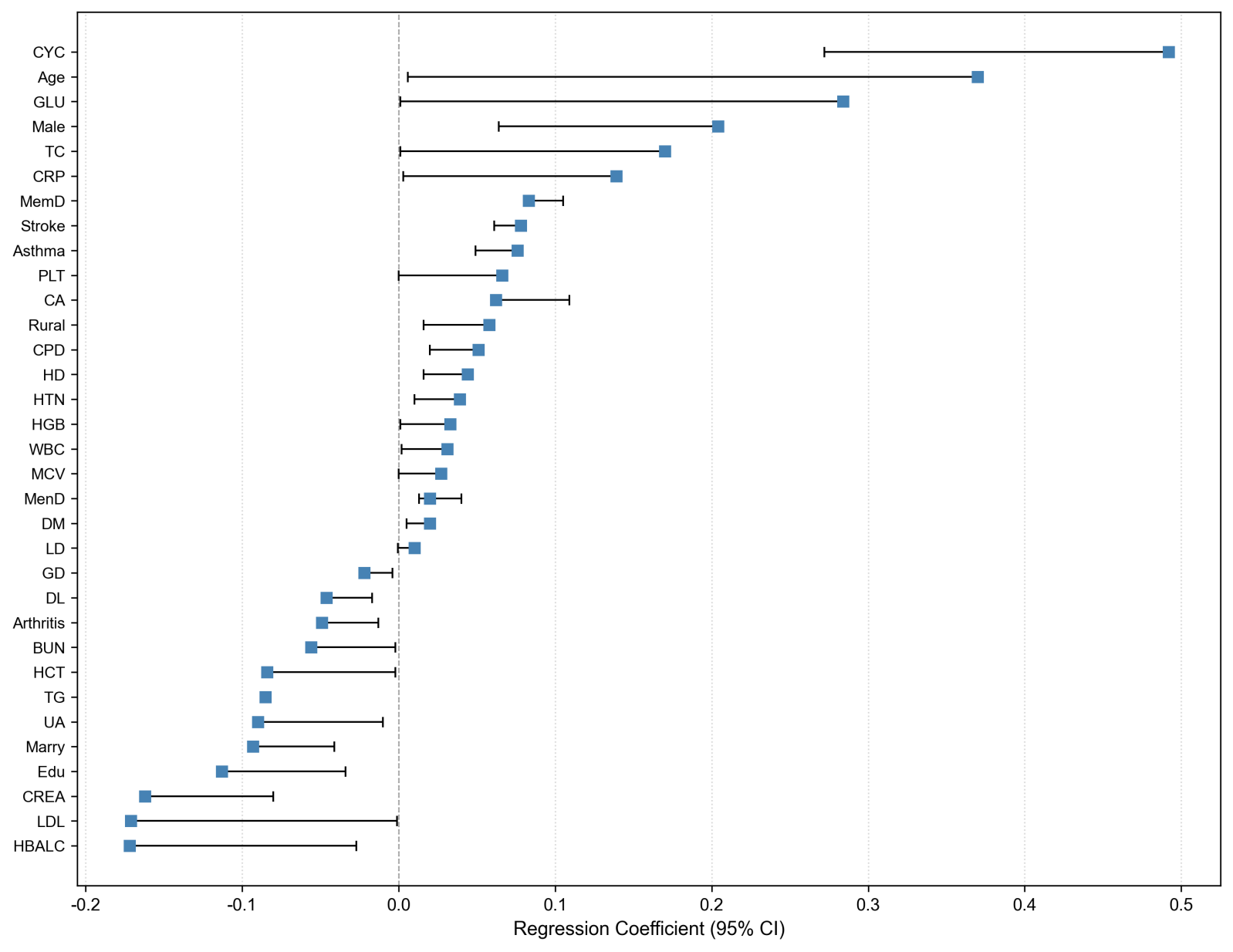

Supplement: S3 Fig — (TIF) [file pdig.0001528.s005.tif]
