# Supplementary material for: A digital marker for stratifying cardiovascular metabolic comorbidities among the middle-aged and elderly adults
Source: PLOS Digit Health. 2026 Jul 2;5(7):e0001528. doi: 10.1371/journal.pdig.0001528 (PMC13327254; doi:10.1371/journal.pdig.0001528)
Supplement: Attachment — Submitted filename: renamed_88607.docx [file pdig.0001528.s007.docx]

Submission ID: PDIG-D-26-00009

First Author: Dr Danhui Mao

Index of Change

Dear editors and reviewers :

Thank you for your valuable comments on our manuscript. We attached great importance to the concerns raised by the editors and reviewers regarding the insufficient statistical methods, and carefully analyzed each piece of feedback, making comprehensive revisions to the statistical approach and study design. To present the modifications clearly and accurately, we have provided point-by-point responses to each comment below. Additionally, we invited a native English speaker to review this letter to ensure that the language is formal and accurate.

| **Comments** | **Review Comments to the Editor** |
| --- | --- |
| **Editors** | The manuscript addresses a clinically relevant topic regarding the use of computational methods and digital markers for health risk stratification. However, as noted by two reviewers, the statistical analysis is not sufficiently rigorous, and there are fundamental methodological concerns (circularity, information leakage, mismatch between study design and claims) that prevent the manuscript from meeting the journal’s publication criteria in its current form. |
| **Response** | We sincerely thank you and the reviewers for your in-depth critique and guidance on our manuscript. You raised several fundamental issues. These include circular reasoning, information leakage, and the mismatch between the study design and our claims. We have made revisions point by point according to the specific comments from the three reviewers.  Specifically, we have clarified the digital marker as a continuous measure. We strictly defined the stratification as cross-sectional based on percentiles. In the Discussion, we acknowledge that its clinical utility requires further validation against independent endpoints. We have distinguished the two-step process of regression and classification. We also supplemented confidence intervals, calibration curves, and decision curve analysis. Through sensitivity analysis, we have shown that the marker does not rely on a single disease proxy.  Furthermore, we have thoroughly removed inappropriate terms such as "dynamic progression" and "causal." We replaced "stage" with neutral grouping terminology. We uniformly revised "causal" to "structural." In addition, we have further refined the logical framework of the Introduction and Discussion to make them clearer and more rigorous.  The revised manuscript has achieved substantive improvements in methodological rigor and transparency of conclusions. We now submit it for your consideration.  Thank you once again for your assistance. |

| **Comments** | **Review Comments to the Author** |
| --- | --- |
| **Reviewer # 1** | Thank you for the submission. The idea—building a digital marker to help stage cardiovascular–metabolic multimorbidity (CMM) in middle-aged and older adults—fits the scope of PLOS Digital Health and could be clinically useful. That said, I recommend Major Revision because several "foundation-level" items are currently not clear enough (or not validated strongly enough) to support the manuscript’s main claims. In plain terms: right now I cannot tell (i) what the proposed "stages" truly mean clinically, and (ii) whether the modeling and evaluation are set up in a way that rules out shortcuts, leakage, or over-interpretation.  1)What exactly is being "staged," and is it more than a renamed disease count?As written, the workflow appears to train models to predict disease count and then use the resulting marker to assign Early/Middle/Late stages. This risks circularity: if "stage" is derived from disease count thresholds (directly or indirectly), then the marker may simply reproduce the same information in a more complex form. Please define clearly: (a) the exact diseases included in "disease count," diagnostic criteria and time window; (b) how Early/Middle/Late labels are created (fixed cutoffs? data-driven clustering? clinical rationale?); and (c) why these stages reflect clinically meaningful progression rather than a convenient binning. Critically, please demonstrate added value beyond simple disease count stratification—for example by linking stages to independent outcomes (future disease accrual, hospitalization, cardiovascular events, mortality) or other clinically accepted endpoints. |
| **Response** | Thank you very much for your thorough review and valuable comments. We fully understand the two core issues you raised: first, whether the definition of "stage" is essentially just another way of expressing disease count, and second, whether the modeling and evaluation process involves circular reasoning or the risk of overinterpretation. In response to these concerns, we elaborated in the Introduction and Discussion that the proposed digital marker is fundamentally a continuous measure of the overall status of CMM. This indicator integrates multiple clinical laboratory parameters and demographic characteristics to reflect the holistic state of coexisting multimorbidity, rather than simply serving as a substitute for disease count. Our classification into Early/Middle/Late stages is based on the distribution of this continuous marker and is intended to facilitate clinical stratification and the development of intervention strategies, rather than merely renaming disease counts. Then, we added a clarification in the Discussion that although the current staging is derived from the predicted disease count, its clinical value lies in integrating multiple indicators to systematically identify the progression stages of CMM.  The specific revisions and their locations are detailed below. |
| **Changes in the Paper** | In response to the three detailed issues raised by the reviewer, we have made revisions to the original text as follows:  First, to address **issue** **(a)** regarding the specific diseases included in the disease count, the diagnostic criteria, and the time window, we revised the original sentence in ***Methods 2.2*** ***from*** "The presence of chronic diseases was defined by a physician-diagnosed history of hypertension, diabetes mellitus, heart disease, stroke, dyslipidemia, chronic kidney diseases, cancer, chronic pulmonary diseases, mental disorders, arthritis, liver diseases, gastrointestinal diseases, asthma, or memory disorders." ***to*** "his study included 14 chronic conditions for analysis: hypertension, diabetes, heart disease, stroke, dyslipidemia, chronic kidney disease, cancer, chronic lung disease, mental disorders, arthritis, liver disease, gastrointestinal disease, asthma, and memory disorders. The presence of each chronic condition was determined based on self-reported physician diagnosis in the China Health and Retirement Longitudinal Study (CHARLS) questionnaire, which records whether a participant had ever been diagnosed with the condition by a doctor. The diagnostic information reflects cumulative prevalence up to the survey year and was used to determine the overall multimorbidity burden during the study period."  Second, to address **issue (b)** concerning how the Early, Middle, and Late stage labels were created, we revised the original sentence in ***Methods 2.4*** ***from*** "Subsequently, patients were stratified into three ordered disease stages based on the percentiles of the digital marker: Early Stage (≤*P_33_*), Middle Stage (between *P_33_* to *P_67_*), and Late Stage (>*P_67_*)." ***to*** "The digital marker was defined as the disease count predicted by the optimal machine learning model, serving as a continuous measure of systemic multimorbidity burden. Based on the distribution of this marker, patients were stratified into three ordered stages: early (≤*P_33_*), middle (between *P_33_* to *P_67_*), and late (>*P_67_*). This staging integrates multiple clinical and demographic characteristics as a composite indicator to enhance clinical interpretability and support stage-based intervention."  Third, to address **issue (c)** regarding the clinical meaning of the stages and their added value beyond disease count, we ***added*** the following statement to the fourth paragraph of the ***Discussion*** (preceding the original section "The analysis of CMM network stages..."): "Although the three stages were derived from the predicted disease count, the digital marker integrates multidimensional clinical laboratory and demographic information, thereby capturing systemic pathophysiological states beyond simple disease enumeration. To further demonstrate its clinical utility beyond disease count stratification, the future studies can evaluate its association with independent clinical endpoints such as hospitalization, cardiovascular events, and mortality. Such validation will establish the stage definition as a clinically meaningful progression indicator rather than a convenient statistical binning." In addition, we revised the original sentence in the ***Conclusion*** ***from*** "The digital marker generated based on these key clinical laboratory parameters and demographic characteristics can robustly and effectively describe the evolutionary stages of the CMM network." ***to*** "By integrating key clinical laboratory parameters and demographic characteristics, the digital marker provides a continuous and multidimensional characterization of the systemic state of chronic disease multimorbidity. The three stages delineated based on this marker may serve as a practical tool for depicting disease progression, with potential applications in early identification and stage-based intervention. In comparison to classification based solely on disease count, this marker demonstrates certain advantages in integrating multidimensional information."  Thank you again. |

| **Comments** | **Review Comments to the Author** |
| --- | --- |
| **Reviewer # 1** | 2)The prediction task and the reported evaluation metrics do not line up (regression vs classification).  If the primary target is a count (disease count), the natural framing is regression or count modeling; however, the reported metrics (Accuracy/Precision/Recall/F1) are classification metrics. Please clarify the primary endpoint and align the analysis accordingly. If you are doing regression, report MAE/RMSE/R² (or count-model metrics such as Poisson/negative binomial where appropriate). If you are doing classification (e.g., stage classification), specify class definitions and prevalence, report appropriate discrimination metrics (e.g., AUC/PR-AUC as relevant), and include calibration. In either case, provide uncertainty (confidence intervals) via bootstrap or repeated/nested cross-validation rather than a single split. |
| **Response** | Thank you for the valuable comments from the reviewer. We fully agree that the original manuscript had issues with unclear task definitions and indicator reporting, and we will address these carefully in the revised manuscript. Regarding the mismatch between the prediction task and the reported evaluation metrics, our study design consists of two steps: the first step is a regression task, in which ten machine learning algorithms are used to predict the number of diseases per individual based on key clinical laboratory indicators and demographic characteristics, generating a continuous digital marker to quantify the burden of multimorbidity. In the second step, based on the tertiles of this digital marker (≤*P_33_*, *P_33_*–*P_67_*, >*P_67_*), the population is divided into three ordered stages (Early, Middle, and Late) forming a three-class classification task. Therefore, Table 1 and Figure 6 present the performance of this classification task, and reporting accuracy, precision, recall, and F1-score is appropriate. To more comprehensively address your suggestions, we will: (i) clearly distinguish these two steps in Section 2.4; (ii) supplement discrimination metrics for the classification task (reporting macro-average and weighted-average *AUC* and *PR-AUC* using a one-vs-rest strategy) and calibration metrics (providing calibration curves in the supplementary materials); and (iii) replace single point estimates with 95% confidence intervals for all core metrics (including *MAE*, *RMSE*, and *R^2^* for the regression task) using 5×5-fold cross-validation, and bootstrap resampling (with 1,000 iterations) to ensure transparency in the evaluation of the classification task. Once again, we thank you for your thorough and professional review, and we will carefully revise the manuscript accordingly to enhance its rigor and scientific clarity. The specific revisions and their locations are detailed below. |
| **Changes in the Paper** | We revised the original sentence in the ***2.4 Construction of the digital marker and the CMM staging*** ***from*** "Ten machine learning models, namely RandomForest, XGBoost, MLP, KNN, GradientBoosting, SVR, LinearRegression, Ridge, ElasticNet and Lasso were compared. Using the top 7 network-importance-ranked clinical laboratory parameters and demographic characteristics as input variables, the total number of disease cases was predicted, with the model predictions serving as digital markers," ***to*** "This section consists of two sequential steps. In the first step, ten machine learning algorithms (RandomForest, XGBoost, MLP, KNN, GradientBoosting, SVR, LinearRegression, Ridge, ElasticNet, and Lasso) are applied, using the top seven clinical laboratory parameters and demographic characteristics ranked by network importance as input variables to predict the total number of diseases per individual, thereby generating a continuous digital marker to quantify the multimorbidity burden. In the second step, based on tertiles of this digital marker, the population is stratified into three ordered stages (early, middle, and late), forming a three-class classification task." Accordingly, Table 1 and Figure 6 present the model performance for this classification task, and reporting *Accuracy*, *Precision*, *Recall*, and *F1* score is appropriate. To more comprehensively address the reviewer’s comments, we further ***added*** *AUC and* *PR-AUC* in ***Manuscript Table 1*** . Additionally, we included calibration curves in the ***Supplementary Table 1*** to assess model calibration, and supplemented all core metrics (*MAE*, *RMSE*, and *R^2^*) from the regression task in the first step, and reporting 95% *Confidence Intervals* to replace the original single point estimates.  **Table 1 Regression Performance Comparison of Different ML Algorithms (top 7) (Supplementary)** **Models** \| ***MAE*, 95% *CI*** \| ***RMSE*, 95% *CI*** \| ***R²*, 95% *CI***; ***RandomForest*** \| 0.4494 (0.4418–0.4575) \| 0.5790 (0.5677–0.5901) \| 0.8668 (0.8637–0.8697); ***XGBoost*** \| 0.6591 (0.6467–0.6713) \| 0.8726 (0.8544–0.8903) \| 0.6975 (0.6867–0.7074); ***MLP*** \| 0.8763 (0.8606–0.8916) \| 1.1325 (1.1097–1.1532) \| 0.4904 (0.4718–0.5072); ***KNN*** \| 1.0371 (1.0177–1.0545) \| 1.3438 (1.3178–1.3688) \| 0.2826 (0.2670–0.3000); ***GradientBoosting*** \| 1.1322 (1.1117–1.1516) \| 1.4496 (1.4197–1.4775) \| 0.1652 (0.1518–0.1781); ***SVR*** \| 1.0824 (1.0610–1.1037) \| 1.4828 (1.4493–1.5149) \| 0.1265 (0.1099–0.1437); ***LinearRegression*** \| 1.1890 (1.1683–1.2102) \| 1.5249 (1.4944–1.5553) \| 0.0762 (0.0651–0.0870); ***Ridge*** \| 1.1890 (1.1683–1.2102) \| 1.5249 (1.4944–1.5553) \| 0.0762 (0.0651–0.0870); ***ElasticNet*** \| 1.2027 (1.1815–1.2237) \| 1.5351 (1.5035–1.5663) \| 0.0638 (0.0557–0.0716); ***Lasso*** \| 1.2118 (1.1906–1.2332) \| 1.5419 (1.5101–1.5735) \| 0.0555 (0.0489–0.0622) Thank you again. |

| **Comments** | **Review Comments to the Author** |
| --- | --- |
| **Reviewer # 1** | 3) High risk of information leakage / "shortcut learning" needs to be explicitly addressed.  Given that key lab markers such as HbA1c/GLU are closely tied to diabetes (and diabetes appears among disease nodes), the model could achieve strong performance by detecting an obvious disease proxy rather than capturing a generalizable multimorbidity staging signal. In addition, the overall pipeline (network construction → feature ranking/selection → model training) is prone to leakage if any step uses the full dataset before train/test separation. Please document a leakage-safe procedure: network learning and feature selection must occur within training folds only (nested CV), not on the entire dataset. I also strongly recommend sensitivity analyses such as: removing high-leakage proxy features (e.g., HbA1c/GLU) and reporting performance drop; or redefining the outcome (if clinically justified) to show the marker is not dominated by a single disease proxy. |
| **Response** | This comment is very interesting. We thank the reviewer for the valuable comments. Regarding the concern about shortcut learning, it should be clarified that HbA1c and GLU serve as more than mere "proxy indicators" for diabetes in our network. As demonstrated by the network constructed using DirectLiNGAM, these two nodes exhibit high out-degree and high betweenness centrality, pointing not only to diabetes but also to various cardiovascular or metabolic diseases such as cardiovascular disease and renal impairment. This precisely reflects the clinical reality of dysglycemia as a systemic driver of multimorbidity. The core mechanisms of CMM are insulin resistance, oxidative stress, and chronic inflammation, and HbA1c and GLU represent the most direct clinical manifestations of these mechanisms. Forcibly excluding these indicators would effectively require the model to predict multimorbidity progression while ignoring the core pathophysiological processes, an approach that is clinically untenable. Furthermore, if any indicator strongly associated with a specific disease were to be regarded as shortcut learning, then lipid profiles linked to cardiovascular disease, or creatinine linked to kidney disease, would also be excluded, ultimately rendering the features selected through network analysis clinically meaningless. The goal of our study is precisely to identify indicators with genuine systemic influence within the multimorbidity network through unbiased network analysis, including HbA1c and GLU, which in itself represents an important finding rather than a methodological flaw.  Regarding the concern about data leakage, we provide the following clarification. First, feature selection was conducted as an unsupervised process based on network centrality measures (e.g., out-degree, betweenness centrality) and did not involve the outcome variable (disease count); therefore, the risk of information leakage from the test set into feature selection is extremely low. Second, the primary objective of this study is to construct a clinically interpretable digital marker based on network topology, rather than to develop a discriminative model for prospective prediction. Although nested cross-validation represents a more rigorous approach, it would lead to inconsistent network structures across folds, making it difficult to obtain stable centrality rankings and clinically meaningful conclusions. Accordingly, we prioritized the stability and interpretability of the network structure in the current study. The machine learning models in this study were used primarily to select an optimal algorithm for generating the digital marker, and the reported metrics such as *Accuracy* and *F1* score were intended mainly for internal consistency assessment rather than for claiming absolute generalizability. We have added a corresponding statement in the ***Limitations*** section of the manuscript, noting that future external validation will further evaluate the generalizability of the marker (see the revised ***Limitations*** section for details).  In addition, to evaluate whether the digital marker relies excessively on metabolic features closely associated with diabetes, we conducted a sensitivity analysis by removing HbA1c and GLU from the feature set and retraining the RandomForest model using the remaining five features (TC, UA, HCT, TG, WBC, CYC). Since the reviewer raised this comment, which is very valuable for discussion. we conducted a sensitivity analysis by removing HbA1c and GLU from the feature set and retraining the RandomForest model. Interestingly, model performance after exclusion was comparable to, or even slightly exceeded, that of the full model. This finding suggests that the predictive signal of the digital marker is not disproportionately dependent on these two metabolic features. Instead, the predictive capacity is distributed across a broader set of network derived features, indicating that the marker captures multimorbidity signals beyond a single disease proxy. This result further supports the robustness of our network based feature selection approach and suggests that while shortcut learning is methodologically important, it does not substantially affect the validity of our digital marker. Detailed results are provided in ***Supplementary Table 2***.    Thank you again. |

| **Comments** | **Review Comments to the Author** |
| --- | --- |
| **Reviewer # 1** | 4)The causal discovery / DAG component (DirectLiNGAM) requires stronger justification and robustness checks.  DirectLiNGAM relies on assumptions (e.g., linear relations, non-Gaussian noise, acyclicity, limited unmeasured confounding) that can be difficult to satisfy in mixed clinical data, especially when combining continuous labs with binary disease indicators. Please provide enough methodological detail for readers to assess appropriateness: variable types/encoding, preprocessing, missing data handling, and any assumption checks. Also, the results should be shown to be stable: edge stability and centrality ranking stability via bootstrap/resampling; sensitivity to preprocessing; and ideally comparison to at least one alternative structure-learning approach. Finally, please use careful language: unless assumptions are convincingly supported, the DAG/edges should be described as structural/associational rather than definitive causal mechanisms. |
| **Response** | Thank you very much for your valuable comments, which provide important guidance for enhancing the rigor and scientific quality of our manuscript. Our initial choice of the DirectLiNGAM algorithm was motivated by the fact that its underlying assumptions align more closely with clinical data compared to alternative methods. DirectLiNGAM decomposes the observed data matrix by identifying a lower triangular permutation matrix (i.e., a causal ordering matrix). Its robustness is reflected in the ability to uniquely determine causal order under the assumption of non-Gaussianity. Specifically, for any two independent non-Gaussian signals, their linear mixture cannot be reduced to independent components via orthogonal transformation, thereby circumventing the issue of equivalent models that arises in traditional structural equation modeling under Gaussian assumptions. The algorithm ensures global uniqueness of the causal order through sequential decomposition of the variable covariance and residual matrices. Clinical laboratory measurements often exhibit skewed distributions, with error terms that typically deviate from Gaussianity. In this study, we did not apply normal transformation to the variables during preprocessing, thereby preserving their original distributional characteristics. This approach helps satisfy the non-Gaussian noise assumption and enhances the identifiability of DirectLiNGAM. DirectLiNGAM employs an iterative procedure of sequential regression and independence testing. In each iteration, the algorithm selects the root node by minimizing the dependence between the residual and the current variable—a process equivalent to projection pursuit within a progressively shrinking linear subspace. The Gram-Schmidt orthogonalization process ensures consistent convergence of the projection direction given sufficient sample size, thereby enhancing the algorithm’s robustness to sampling variability and outliers. Binary variables (e.g., disease status) can be viewed as thresholded manifestations of continuous latent variables. DirectLiNGAM is based on linear models and independent noise assumptions; its matrix factorization framework remains valid when handling mixed continuous and binary variables, as binary variables can be related to continuous variables through a linear probability model, while the independence tests employed (such as kernel-based independence tests) exhibit a degree of insensitivity to the specific forms of variable distributions.  The pathophysiological process of CMM is inherently unidirectional (from risk factor exposure → metabolic abnormality → organ damage → disease onset). Although interactions among diseases may exist, in cross-sectional data we assume that the observed associations reflect cumulative effects rather than instantaneous feedback. The directed acyclic graph (DAG) constructed in this study is based on cross-sectional data; thus, the acyclicity assumption is reasonable for inference at a single time point. While there are indeed numerous nonlinear relationships between clinical laboratory measurements and disease status, empirical evidence suggests that, after controlling for confounders, most biomarkers exhibit approximately linear associations with disease risk, particularly within moderate ranges of variation. In this study, continuous variables were standardized using Z-scores, and the randomness of residuals was verified through model diagnostics. Furthermore, we explicitly acknowledged the limitations of the linearity assumption in the Methods section and discussed them as a study limitation.  This study incorporated demographic characteristics, core clinical laboratory measurements, and disease statuses, which collectively cover key nodes in the development of CMM. Although unmeasured confounding (e.g., genetic factors, environmental exposures) cannot be completely ruled out, the key nodes identified through network analysis (e.g., glucose, lipids, kidney function indicators) are highly consistent with known pathophysiological mechanisms, indirectly supporting the notion that confounding is controlled within an acceptable range. In the revised manuscript, we have supplemented details regarding variable preprocessing, which can be found in the ***Methods 2.3*** and ***Methods 2.5*** section, and we have added a bootstrap-based stability analysis.  Regarding the comparison of alternative methods, we attempted several approaches during the study; however, causal discovery algorithms capable of handling mixed continuous and binary variables are currently very limited, and each method has inherent limitations that make direct application to this study challenging. For example, the PC algorithm (based on conditional independence testing) and the GES algorithm (based on score-based search) both assume that the data follow a multivariate Gaussian distribution, an assumption that is clearly violated in our dataset. Structural learning based on Bayesian networks (e.g., BN-HC) involves extremely high computational complexity and is prone to local optima when the number of variables is large.  With respect to your suggestion to moderate the deterministic use of the term "causal" we have carefully considered this point and decided to adopt more cautious wording. Although some recent literature has used the term "causal" more directly, we believe caution is warranted given the cross-sectional nature of our data. Accordingly, we have uniformly revised "causal network" to "structural network", "causal relationship" to "structural association", and "causal order" to "structural order" throughout the manuscript to more accurately reflect the structural associations captured by the network. Detailed revisions are indicated in the highlighted sections of the manuscript ***Methods 2.3***, ***Methods 2.5***, ***Results 3.2***, ***Limitation*** section, etc.  Thank you again. |

| **Comments** | **Review Comments to the Author** |
| --- | --- |
| **Reviewer # 1** | 5) Claims about "dynamic evolution/progression" must match the study design.  If the study is cross-sectional, it cannot directly demonstrate progression over time; in that case, the manuscript should be reframed as cross-sectional stratification. If longitudinal data exist, please explicitly describe the time structure and validate the "stages" by showing transitions or prediction of future disease accumulation, ideally with out-of-time validation. |
| **Response** | Thank you for your valuable comments. We fully agree with the key issue you raised: cross-sectional designs cannot directly demonstrate dynamic evolution or progression over time. Therefore, we will strictly follow your suggestion to revise the inappropriate wording in the manuscript.  Specifically, we examined each sentence for terms implying longitudinal inference, such as "dynamic evolution," "progression," "over time," "gradually develop into," and "progress to," and replace them with expressions that merely describe cross-sectional status, such as "differences between groups" and "characteristics based on group features." At the same time, we will delete phrases that imply temporal trends and replace them with static descriptions like "observed in the higher scoring group" and "comparisons between groups show." Furthermore, we will replace all instances of "stage" (including "early stage", "middle stage", "late stage", "developmental stage", "evolutionary stage") with neutral terms such as "group", "subgroup", or "stratum" (e.g., "low group", "medium group", "high group") to avoid any unintended implication of temporal progression. Please see the highlighted sections (green color) in the main text for details.  Thank you again. |

| **Comments** | **Review Comments to the Author** |
| --- | --- |
| **Reviewer # 1** | 6) Clinical usefulness is not yet demonstrated; baseline comparisons and validation are needed.  Even if the model predicts disease count well, readers need to know whether it improves on simpler alternatives (age/sex + basic labs; standard statistical models; direct disease-count thresholds). Please add strong baselines and show incremental value. External validation is preferred; if not possible, at least provide rigorous internal validation (nested CV, or time-split validation when appropriate). Consider adding calibration and decision-analytic evaluation (e.g., decision curve analysis) if you want to claim clinical decision support value.  In summary, the manuscript has a potentially valuable direction, but the current version leaves key questions unanswered: what the "stages" mean clinically, whether the evaluation is leakage-safe and appropriately measured, whether the causal/network claims are robust, and whether the marker offers real added value beyond disease count. Addressing the above points with clear definitions, rigorous validation, and reproducible reporting would substantially improve technical soundness and the credibility of the conclusions. |
| **Response** | Thank you for your valuable feedback. We have added comparisons with simple baselines, as detailed in ***Supplementary Table 3***. We fully understand the reviewer's concern regarding the generalizability of our model. Although nested cross-validation can theoretically provide a less biased estimate of the generalization error, its underlying mechanism requires that the entire feature selection process be independently re performed on the training set of each outer fold. In this study, this process refers to centrality calculation and feature screening based on network analysis. This would cause the network structure to fluctuate considerably with changes in the training set. Due to differences in the sample composition of the training sets across outer folds, the association networks constructed by causal discovery algorithms (e.g., DirectLiNGAM) may differ in terms of node connections, edge directions, and centrality metrics (such as out degree and betweenness centrality). Even if the overall samples are drawn from the same distribution, random sampling may lead to certain nodes exhibiting high centrality in some outer folds but not in others. Such cross fold inconsistency makes it difficult to obtain a stable and reproducible centrality ranking, thereby hindering an accurate identification of which indicators truly serve as systemic drivers of multimorbidity. This study aims to identify digital markers with genuine systemic influence, which requires deriving reproducible conclusions based on a fixed network structure. For the above reasons, we prioritized structural stability and clinical interpretability during the network construction stage. Regarding the validation of generalizability, we have already begun to accelerate the construction of an external dataset. It should be noted that building a high quality external dataset requires substantial time and financial resources. To date, although we have collected clinical data from five affiliated hospitals of our university since 2020, the presence of a large number of missing values in these data means that the current amount of accumulated data is still insufficient to support a valid external validation. In addition, we have included calibration curves and decision curve analysis to support clinical decision value, as shown in ***Supplementary Fig. 1.*** Moreover, we note that we have already performed a sensitivity analysis excluding HbA1c and GLU, which can also serve as evidence of robustness. **Table 3 Regression Performance Comparison of Baseline Models and Ours** **Models** \| ***MAE*, 95% *CI*** \| ***RMSE*, 95% *CI*** \| ***R²*, 95% *CI***; ***Ours*** \| 0.4494 (0.4418–0.4575) \| 0.5790 (0.5677–0.5901) \| 0.8668 (0.8637–0.8697); ***Baseline A*** (LinearRegression with Age+Sex+GLU+TC+UA) \| 1.8185 (1.7831–1.8574) \| 2.4362 (2.3788–2.4939) \| 0.1257 (0.1110–0.1387); ***Baseline B*** (LinearRegression with all demographic and labs variables) \| 1.5147 (1.4857–1.5433) \| 2.0106 (1.9606–2.0644) \| 0.4045 (0.3795–0.4259)  **Fig. 1 Decision Curve Analysis for Identifying High Group Patients** We sincerely thank you for your rigorous, insightful, and deeply thoughtful review process. Each of your comments not only helped us identify the limitations of our original manuscript but also prompted us to repeatedly reflect on and systematically restructure our entire research methodology throughout the revision process. Every discussion and revision we undertook has been profoundly inspiring. Your high standards have driven us to continuously refine and improve our feature engineering, model validation, and clinical evaluation. We truly feel that your review comments themselves are a precious academic gift. We believe that the revised manuscript has achieved substantial improvements in both rigor and clinical value. Once again, we extend our heartfelt gratitude for your hard work and professional guidance. Your help has greatly benefited us. Thank you again. |

| **Comments** | **Review Comments to the Author** |
| --- | --- |
| **Reviewer # 2** | Overall, the study addresses a clinically relevant and timely topic aligned with the scope of PLOS Digital Health, and the methodological framework is generally sound.  The rationale for categorizing the continuous digital marker into three stages using tertile-based cut-points (P33 and P67) requires further clarification. While this approach is statistically convenient, the manuscript would benefit from a clearer explanation of how these thresholds relate to clinically meaningful distinctions in cardiometabolic multimorbidity progression. In addition, the robustness of the proposed staging framework could be strengthened by conducting sensitivity analyses using alternative cut-points (e.g., quartiles or clinically informed thresholds) to demonstrate that the observed patterns and conclusions are not highly dependent on the specific binning strategy chosen. |
| **Response** | Thank you for the reviewer’s valuable comments. In this paper, we used quantiles to divide the continuous biomarker into different stages, based primarily on the following considerations. First, there is currently no recognized clinical threshold for this biomarker to guide the staging of cardiometabolic multimorbidity, either nationally or internationally. In this context, the quantile method serves as an objective and reproducible data‑driven grouping strategy, which allows subjects to be divided into stages with approximately equal sample sizes according to the distribution of the biomarker in the study population. This approach not only avoids the subjectivity inherent in artificially defined cut points, but also ensures that each subgroup has sufficient statistical power for subsequent comparisons, making it particularly suitable for risk stratification in exploratory research. Second, although different quantile options (e.g., medians, tertiles, or quartiles) are statistically feasible, our final choice of tertiles was not based solely on statistical convenience. During the data exploration phase, we systematically compared the baseline clinical characteristics of the groups defined by different quantile splits. The results showed that when tertiles (low, medium, and high groups) were used, most of the major clinical indicators exhibited a clear, monotonic, stepwise trend as the biomarker level gradually increased, and the differences between groups were statistically significant (*P* < 0.05). In contrast, the median split was too coarse to effectively distinguish the intermediate‑risk population. Although quartiles provide a more detailed stratification, the differences in clinical characteristics between some adjacent groups were not obvious in our study, which reduced the parsimony and clinical interpretability of the staging framework. Therefore, tertiles, while maintaining sufficient discriminative ability, also achieve parsimony in staging and are more suitable as the primary grouping strategy for this study. Furthermore, to assess the robustness of the proposed staging framework, we performed sensitivity analyses using quartiles (*P_25_*, *P_50_*, and *P_75_*) as alternative cut points. The results were consistent with those obtained from the tertile‑based group (see ***Table 4*** for more details ndicating that our subgroup analysis framework has good robustness.    Thank you again. |

| **Comments** | **Review Comments to the Author** |
| --- | --- |
| **Reviewer # 2** | Although the reported model performance metrics (accuracy, precision, recall, and F1 score) are acceptable for comparing algorithms, the absence of calibration assessment limits evaluation of the model’s clinical applicability. Reporting calibration measures, such as calibration plots, would provide important insight into how well predicted disease burden aligns with observed outcomes across the range of the digital marker. Including such analyses would enhance the interpretability and potential translational value of the proposed digital marker in real-world clinical or population health settings. |
| **Response** | Thank you for your thorough consideration of the importance of calibration in assessing clinical applicability. As you pointed out, reporting only accuracy, precision, recall, and F1 scores, while acceptable for algorithm comparison, may limit the interpretability and translational value of the model in real-world clinical or population health settings. We fully agree with this perspective. To enhance the interpretability and translational value of the proposed digital marker in real-world clinical or population health environments, we have made the following targeted revisions. First, for all core performance metrics (including accuracy, precision, recall, F1 score, AUC, PR-AUC, etc.), we have supplemented the reporting with 95% confidence intervals derived from 5×5-fold cross-validation combined with bootstrap resampling (1,000 iterations), in order to more comprehensively reflect model uncertainty. Details are presented in ***Table 1*** of the ***Main text*** and ***Tables 1-4*** in the ***Supplementary*** materials. Second, we have added a decision curve analysis (see ***Supplementary Fig. 1***) to evaluate the net clinical benefit of the digital marker across different threshold probabilities. These revisions will effectively strengthen the foundation for evaluating the clinical applicability of our model.  Thank you again for your guidance. |

| **Comments** | **Review Comments to the Author** |
| --- | --- |
| **Reviewer # 3** | General comment:  The manuscript is difficult to follow in its current form. The overall purpose of the paper is not sufficiently clear. It remains ambiguous whether the authors aim to present a methodological study (as suggested in the discussion) or to emphasize the clinical relevance of their analysis (as stated in the introduction). I recommend clearly focusing on one of these objectives.  The manuscript would benefit from more streamlined writing. At present, methodological details appear in the background section, while the discussion does not sufficiently elaborate on the clinical implications of the findings. In addition, the results presented do not fully support the conclusions drawn.  Major comments:  Introduction  The first sentence raises several questions. It may not be obvious to readers why CMM is described as a complex disease, nor why there is such a marked difference in prevalence between China and the Middle East/North Africa. What about other regions of the world, and what is the rationale for focusing exclusively on these areas? More generally, I suggest revising the opening section of the introduction, as it currently presents a sequence of facts without a clear narrative or defined objective.  In the final paragraph of the introduction, I recommend focusing more explicitly on the study objectives and moving detailed methodological information to the Methods section. |
| **Response** | We greatly appreciate the valuable comments and suggestions from the reviewers. After careful consideration, we fully concur with your assessment and have thoroughly reorganized and revised the logical framework of the Introduction, which now consists of four paragraphs. The first paragraph describes the research background and significance of CMM as a complex disease and presents its current prevalence. The second paragraph systematically reviews the academic development trajectory, illustrating how researchers’ understanding of CMM has evolved from single diseases to multimorbidity and from simple associations to complex relationship networks. The third paragraph identifies the core limitation of existing research methods—namely, that current approaches cannot effectively characterize the complex relationships among comorbidities—and thereby proposes an urgent need to adopt complex network methods to describe such relationships in CMM. The fourth paragraph states the research hypothesis and core objectives of this study. In parallel, we have moved the detailed methodological information originally presented in the Introduction to the Methods section, and have supplemented the Discussion with an elaboration of the clinical implications of our findings. Details are presented in ***Manuscript.***  Thank you again for your guidance. |

| **Comments** | **Review Comments to the Author** |
| --- | --- |
| **Reviewer # 3** | Methods  What is the rationale for comparing ten different machine learning models?  How were repeated measurements handled for patients who contributed data from two waves? |
| **Response** | Thank you for your valuable comments. To avoid over-sensitivity of any single model to specific data distributions or noise, and to enhance the robustness and generalizability of our conclusions, we systematically compared the performance of these models in the prediction task of this study. The ten machine learning models were selected because they are among the most commonly used models in clinical research. Clinical data typically encompass linear trends, nonlinear interactions, high-dimensional sparse features, and moderate multicollinearity. The underlying assumptions of the selected models align well with the actual characteristics of clinical data. For instance, linear regression assumes error independence, tree-based models are capable of capturing non-monotonic relationships, and regularization methods are well-suited to handling multicollinearity.  Thank you for raising the concern regarding repeated measurements. Thank you for your correction. After cleaning the data across five waves, we found that most participants took part in only one of the waves, while a small number of participants took part in both Wave 1 and Wave 3. To avoid repeated sampling, this study only included participants who had taken part in exactly one wave as the analytical sample.  Thank you again. |

| **Comments** | **Review Comments to the Author** |
| --- | --- |
| **Reviewer # 3** | Results  There are too many figures and tables, and several are difficult to read without magnification. I recommend selecting a smaller number of key figures and summarizing the remaining results in the text. For example, Figure 4 adds limited value in its current form. The heatmap is difficult to interpret, and without sufficient explanation, its purpose remains unclear. |
| **Response** | Thank you very much for your valuable comments. We fully agree with your observation that the original manuscript contained an excessive number of figures, some of which were difficult to read without magnification. Following your suggestion, we have streamlined the figures and retained only the key graphics that best illustrate the core findings. Specifically, in accordance with your advice, ***Fig. 4*** and the related heatmap (***Fig. 5***) have been moved from the main text to the supplementary materials for interested readers to consult. We greatly appreciate your thorough review and constructive suggestions.  Thank you again. |

| **Comments** | **Review Comments to the Author** |
| --- | --- |
| **Reviewer # 3** | Regarding Table 2, I do not understand the rationale for presenting relative frequencies within categories of a single variable. Would it not be more informative to present relative frequencies across all stages?  Given the sample size, statistically significant associations in multiple linear regression are not unexpected. However, it remains unclear whether the observed effects are clinically meaningful. |
| **Response** | Thank you very much for your valuable comments. We fully understand your concern regarding the presentation of the table. First, the primary purpose of this table is to clearly describe the internal baseline characteristics of the different CMM disease subgroups (low, middle, and high groups). Therefore, we chose to present the proportion of each category within every subgroup, allowing readers to intuitively grasp the population profile of each subgroup. Second, because the subgroup classification was based on percentiles of the digital marker score, which itself carries richer information, we further examined the differences in this score across populations with various demographic, disease, and blood/urine test characteristics. Finally, to streamline the number of tables and avoid information fragmentation, we combined descriptive distributions and statistical inference results in the same table, striving to present the most critical comparative information within the limited space. We once again appreciate your guidance and hope that the above explanation clarifies our considerations.  And thank you for your insightful consideration of the evaluation of clinical applicability. To enhance the interpretability and translational value of the proposed digital marker in real-world clinical or population health settings, we have supplemented all core performance metrics of the machine learning model with 95% confidence intervals (see ***Table 1*** in the ***main text*** and ***Tables 1–4*** in the ***Supplementary***). In addition, we have added a decision curve analysis (see ***Supplementary Fig. 1***) to assess the net clinical benefit of the digital marker across different threshold probabilities. These additions aim to further improve the evaluation of clinical applicability in this paper.  Thank you again for your guidance. |

| **Comments** | **Review Comments to the Author** |
| --- | --- |
| **Reviewer # 3** | Discussion  I did not gain the impression that the paper clearly identifies the core driving factors underlying the evolution of the CMM network. The clinical message should be articulated more clearly.  In addition, the discussion repeats several aspects of the results across different sections. I suggest first discussing the main findings concisely and then placing them into a broader context. This would help distinguish which points stem directly from your results and which are supported by the existing literature. |
| **Response** | We are very grateful to the reviewer for your thoughtful guidance on the logical structure of our writing. The issues you raised prompted us to re-examine the overall organization of the manuscript. Inspired by your insights, the main thread of the paper has become clearer, and the quality of the writing has greatly improved. We sincerely appreciate your valuable comments. In the Discussion section, we have reorganized the content. Specifically, we first provide a concise summary of the core findings, clearly identifying the key driving factors in the evolution of the CMM network. We then place the results in a broader literature context, clearly distinguishing which conclusions are directly derived from our network analyses and which are supported by existing studies. At the same time, we have removed descriptions that were repetitive of the Results section, thereby making the presentation of clinical information more focused and clearer. Details are presented in ***Manuscript.***  Thank you again for your guidance. |

| **Comments** | **Review Comments to the Author** |
| --- | --- |
| **Reviewer # 3** | Minor comments:  Line 97: Missing space between two words.  Line 201: Missing space between two words.  Line 202: Missing space between two words.  Figure 1: What is the rationale for displaying boxes for non-existent participants with more than two waves of data? Additionally, why does the top box state "with 5 fives," which does not appear to be correct for the analyzed sample? |
| **Response** | Thank you for your correction. After cleaning the data across five waves, we found that most participants took part in only one of the waves, while a small number of participants took part in both Wave 1 and Wave 3. To avoid repeated sampling, this study only included participants who had taken part in exactly one wave as the analytical sample. The participants in the blue box in the bottom row of Fig. 1 are our study subjects, while those in the gray box are the participants we excluded.    And the missing space between two words in Line 97 , Line 201 , and Line 202 were added.  Thank you again. |

| **Comments** | **Review Comments to the Author** |
| --- | --- |
| **Reviewer # 3** | Lines 129–131: Please add a reference.  Line 179: XGBoost is mentioned twice. |
| **Response** | Thank you for pointing out the detailed error. We added a reference for the *DirectLiNGAM* algorithm at Lines 129-131. And have removed the redundant *XGBoost* at line 179.  Thank you again. |

| **Comments** | **Review Comments to the Author** |
| --- | --- |
| **Reviewer # 3** | Line 182: How were the thresholds determined? |
| **Response** | Thank you for your question. The penalty coefficients (1.0, 0.1, 0.1) for the *Ridge*, *Lasso*, and *ElasticNet* models mentioned in Line 182 were obtained through a systematic parameter optimization process. Specifically, we employed grid search combined with 5‑fold cross‑validation, aiming to minimize the *mean squared error* (*MSE*) on the training set to determine the optimal regularization strengths. First, a set of candidate α values was defined on a logarithmic scale for each model (e.g., 0.01, 0.05, 0.1, 0.5, 1.0, 2.0, 5.0, 10.0). Then, for each candidate *α*, we performed 5‑fold cross‑validation and calculated the average *MSE*. Finally, the α that yielded the smallest average *MSE* was selected as the final parameter. Following this procedure, the optimal *α* was 1.0 for *Ridge*, and 0.1 for both *Lasso* and *ElasticNet*. In addition, we verified that these optimal values maintain a low *MSE* in their neighbourhood, indicating the stability of the parameter selection. To make the presentation in the paper more accurate and clear, we have revised the original sentence ***from*** "For linear models, *Ridge regression*, *Lasso* and *ElasticNet* were set with regularization strengths of 1.0, 0.1 and 0.1, respectively." ***to*** "For linear models, the regularization strengths of *Ridge regression*, *Lasso* and *ElasticNet* were set to 1.0, 0.1 and 0.1, respectively, which were determined by grid search with cross-validation on the training set."  Thank you again for your careful review. |

| **Comments** | **Review Comments to the Author** |
| --- | --- |
| **Reviewer # 3** | Line 183: Why were five nearest neighbours chosen? |
| **Response** | Thank you for your question. The choice of 5 nearest neighbors in the K-Nearest Neighbors model was based on performance validation on the training set. We evaluated several candidate values, including (*ƙ* = 3, 5, 7, 9), and compared their mean squared error using 5-fold cross-validation. The results showed that *ƙ* = 5 achieved the best balance between bias and variance while being less sensitive to noise in the training data. Moreover, *ƙ* = 5 is a commonly used default value in the field, which facilitates comparison with other studies. Therefore, we ultimately selected five nearest neighbors. To make the presentation in the paper more accurate and clear, we have revised the original sentence ***from*** "*Support Vector Regression* utilized a radial basis function kernel, *K-Nearest* Neighbors employed 5 nearest neighbors, and the *Multilayer Perceptron* was configured with a two-layer hidden structure comprising 100 and 50 neurons, respectively. " ***to*** "*Support Vector Regression* utilized a radial basis function kernel, *K-Nearest* Neighbors employed 5 nearest neighbors (selected via cross-validation), and the *Multilayer Perceptron* was configured with a two-layer hidden structure comprising 100 and 50 neurons, respectively."  Thank you again. |

| **Comments** | **Review Comments to the Author** |
| --- | --- |
| **Reviewer # 3** | Line 209: Consider rephrasing to "included in this analysis." |
| **Response** | Thank you for your suggestion. In accordance with your comment, we have revised the wording in Line 209 ***from*** "included in this paper" ***to*** "included in this analysis" to make the expression clearer and more precise. Thank you again for your careful review.  Thank you again. |

| **Comments** | **Review Comments to the Author** |
| --- | --- |
| **Reviewer # 3** | Line 210ff: I suggest rounding all numbers to one decimal place. |
| **Response** | Thank you very much for your valuable suggestion. Regarding the issue of retaining more decimal places from Line 210 onward, we would like to respectfully explain the reason as follows: since the performance metrics of some algorithms are indeed very close, we were concerned that rounding all values to one decimal place might inadvertently obscure these subtle yet objective differences, thereby affecting readers’ accurate understanding of the comparative performance of different methods. Therefore, we have taken the liberty of temporarily keeping the original decimal precision in the hope of better ensuring the transparency and comparability of the results. Of course, we fully respect your opinion, and if the journal has a uniform formatting requirement, we would be happy to make the adjustments accordingly.  Once again, we sincerely thank you for your thorough review and guidance. |

| **Comments** | **Review Comments to the Author** |
| --- | --- |
| **Reviewer # 3** | Line 225: Use "married" instead of "Marry." |
| **Response** | Thank you for catching this error. We have replaced "Marry" with "married" on Line 225 and have also carefully checked and corrected any similar issues throughout the manuscript.  Thank you again. |

| **Comments** | **Review Comments to the Author** |
| --- | --- |
| **Reviewer # 3** | Table 2: For binary variables, consider presenting only one category. Also, instead of "didn’t have"/"have," use "history of …" |
| **Response** | Thank you for your valuable comments. In accordance with your suggestions, we have modified Table 2 to present only one category for each binary variable (e.g., "history of ..."), and have uniformly replaced the expressions "didn't have" / "have" with "history of ..." throughout the manuscript. We have also carefully reviewed the entire text to ensure that all similar issues have been corrected.  Thank you again for your thorough review. |

| **Comments** | **Review Comments to the Author** |
| --- | --- |
| **Reviewer # 3** | Lines 294 and 304: Is this statement based on your results or on the literature? Please clarify the source and provide a reference if applicable. |
| **Response** | Thank you for pointing out the lack of clarity regarding the source of the statements in Lines 294 and 304. Your comment is very insightful, and we fully agree. Upon re-examination, we recognize that these two statements were indeed based on both our own experimental results and previously published literature; however, the original wording was not sufficiently clear, which could easily lead to misunderstanding.  To address this issue, we have carefully revised the entire manuscript (particularly Lines 294, 304, and other similar passages) in an effort to clearly distinguish between the "findings from the present study" and the "results reported in the literature," and have reorganized the relevant sentences accordingly.  Thank you again. |
