# Supplementary material for: A digital marker for stratifying cardiovascular metabolic comorbidities among the middle-aged and elderly adults
Source: PLOS Digit Health. 2026 Jul 2;5(7):e0001528. doi: 10.1371/journal.pdig.0001528 (PMC13327254; doi:10.1371/journal.pdig.0001528)
Supplement: Attachment — Submitted filename: renamed_8a8b9.docx [file pdig.0001528.s008.docx]

Submission ID: PDIG-D-26-00009

First Author: Dr Danhui Mao

Index of Change

Dear editors and reviewers :

Thank you very much for handling our manuscript and for the insightful and constructive comments from the reviewers. We have carefully considered each point and have made substantial revisions accordingly. We would particularly like to highlight that, beyond addressing formatting, language, and reference issues, we have fundamentally restructured the methodological framework and core conclusion of our study: the prediction target of the digital marker has been shifted from disease count to probability of death. Correspondingly, we introduced an independent test set, nested cross validation, a probability based evaluation metric suite (*AUC*, *PR‑AUC*, *Brier Score*, *Log Loss*), and a systematic comparison of five baseline models. We believe these revisions thoroughly resolve the key weaknesses present in the original submission, including circular reasoning, unclear clinical relevance, risk of information leakage, and an ambiguous evaluation framework. In addition, we performed bootstrap stability analysis for the network structure, streamlined and improved all tables, and carefully edited the language of the entire manuscript. We are fully aware that no study is without limitations; nonetheless, we have approached each comment with the utmost respect for scientific rigor and a persistent pursuit of higher quality, striving to make our work substantially stronger in methodology, clinical interpretability, and credibility of findings. Once again, we sincerely thank the editors and reviewers for their invaluable time and thoughtful guidance. We look forward to the possibility of a positive decision from you.

| **Comments** | **Review Comments to the Editor** |
| --- | --- |
| **Editors** | Please submit your revised manuscript by Jul 25 2026 11:59PM. … Please include the following items when submitting your revised manuscript: * A letter that responds to each point raised by the editor and reviewer(s). * A marked-up copy of your manuscript that highlights changes made to the original version. * An unmarked version of your revised paper without tracked changes. … Please ensure that your Ethics Statement is available in its entirety at the beginning of your Methods section… |
| **Response** | We sincerely thank the Editor for your clear guidance and patient support. We fully understand the importance of proper manuscript formatting and a complete ethics statement for academic publication. Each of your requirements reminds us to treat every detail with the utmost rigour. We have carefully prepared the three required files as instructed. We have also placed the complete ethics statement at the beginning of the Methods section. This process has taught us that scientific research requires not only innovative discoveries but also standardised and transparent presentation. Thank you again for your meticulous guidance. We have learned a great deal from it. |
| **Changes in the Paper** | The specific modifications are as follows. First, we prepared three files. These are the response letter (Response to Reviewers.pdf), the marked‑up manuscript (Revised Manuscript with Track Changes.docx), and the clean version of the manuscript (Manuscript.docx). Second, at the end of Methods section 2.1, the ethics statement now reads in full as follows. "This paper protocol was reviewed and approved by the Ethics Committee of Shanxi Medical University (Approval No: 2025GLL033)… All survey participants provided written informed consent." Third, all authors have verified and confirmed their respective ORCID iDs in the submission system. Fourth, all figure files have been checked using the journal's NAAS tool and meet the resolution and format requirements.  Thank you again. |

| **Comments** | **Review Comments to the Author** |
| --- | --- |
| **Reviewer # 1** | My main concern is that the proposed digital marker is still too closely tied to disease count. The marker is defined as the disease count predicted by the machine learning model, and the Low/Middle/High groups are then created by splitting this predicted marker into tertiles. This does not yet show that the marker captures a clinically meaningful state beyond disease enumeration. |
| **Response** | We are truly grateful to the reviewer for astutely identifying this core issue of circular reasoning. Your criticism has led us to deep reflection and made us realise the flaw in our original manuscript, where the prediction target was conflated with the stratification basis. It is precisely your rigorous guidance that prompted us to fundamentally re‑examine and redefine the digital marker, shifting its prediction target from “disease count” to “probability of death”. This change not only breaks the original circular logic but also directly anchors the digital marker to an independent, objective, and clinically meaningful hard endpoint, namely death. Furthermore, we constructed five baseline models (including a simple disease count model) for systematic comparison, objectively demonstrating the substantial improvement in independent prognostic information provided by our marker. Your every critique has deepened our understanding and respect for scientific rigour. Once again, we sincerely thank you for your meticulous guidance; we have greatly benefited from it. |
| **Changes in the Paper** | In the first paragraph of ***Methods Section 2.4***, we changed the original wording from “predicting disease count” to “predicting the probability of death for each individual”. All related descriptions in the ***Abstract, Results Section 3.4***, and the ***Discussion*** have been revised accordingly. After revision, the mean values of the digital marker changed from 0.72, 1.56, and 3.02 (on the disease count scale) to 0.016, 0.058, and 0.296 (on the mortality probability scale). This change is clearly presented at the end of ***Results Section 3.4*** and in ***Table 2***.  We constructed five baseline models with increasing complexity. These models are M1 (simple disease count), M2 (age + sex), M3 (M2 + basic biochemistry including GLU, TC, and UA), M4 (full variable logistic regression), and M5 (our Ridge model). The key results are as follows. M1 achieved an *AUC* of only 0.589 (95% CI: 0.558–0.620), whereas M5 achieved an AUC of 0.852 (95% CI: 0.824–0.875). The *PR‑AUC* of M1 was 0.147, while that of M5 was 0.530. The *Brier Score* decreased from 0.125 (M1) to 0.078 (M5). These metrics clearly demonstrate that our marker provides independent prognostic information far beyond simple disease count.  The original Table 1 (containing Accuracy, Precision, Recall, and F1) and Figure 4 (confusion matrix) have been completely removed. They have been replaced by a new Table 1, which only includes *AUC, PR‑AUC, Brier Score, Log Loss, Pseudo R²*, and their 95% Confidence Intervals. Descriptions such as “three‑class classification task” have also been deleted and replaced with “probability of death prediction”.  We stratified the data by year of enrolment and randomly split them into a global training set and a global test set at a 7:3 ratio. The test set was completely isolated from data preprocessing, network construction, feature selection, and hyperparameter optimisation, and was used only for final evaluation. Inside the training set, we applied 5×5 nested cross‑validation. Within each fold, missing value imputation (using the median), standardisation, and feature selection were fitted on the training subset and then applied to the corresponding validation subset. Relevant descriptions have been added to ***Methods Section 2.4***.  In ***Results Section 3.4***, we reported the high consistency between the actual mortality rate in the test set (0.122) and the mean predicted probability of the digital marker (0.124). We also added a calibration curve (Supplementary Figure S1) to further demonstrate the accuracy of the digital marker as a measure of mortality risk.  Thank you again. |

| **Comments** | **Review Comments to the Author** |
| --- | --- |
| **Reviewer # 1** | The manuscript also still lacks independent validation of clinical value. The authors state that future studies may evaluate hospitalization, cardiovascular events, and mortality. Those are exactly the outcomes needed to support the proposed clinical use of the marker. Without such validation, claims about early identification, clinical decision support, and subgroup-based intervention should be substantially reduced. |
| **Response** | We are very grateful to the reviewer for this rigorous reminder. We fully agree that any claim regarding clinical utility must be built upon evidence directly linked to patient‑important outcomes. Your criticism made us realise that some of the statements in our original manuscript were indeed overly ambitious and lacked sufficient empirical support. To address this, we have directly defined the digital marker as the predicted probability of death, thereby establishing a direct link between the clinical meaning of the marker and a hard endpoint (mortality). At the same time, we have substantially moderated the overly ambitious claims in the original manuscript, adopted a more cautious and objective tone, and explicitly acknowledged the lack of external validation as a limitation. Your high standards have taught us how to exercise appropriate restraint and rigour in scientific research, which will serve as invaluable guidance for our future work. |
| **Changes in the Paper** | In the Abstract, ***Methods Section 2.4***, and ***Results Section 3.4***, we explicitly set the prediction target as "probability of death". In the ***Results***, we reported the high consistency between the actual mortality rate (0.122) and the mean predicted probability (0.124). We also added a calibration curve (***Supplementary Fig. S1***), which shows that the predicted probabilities closely agreed with the observed outcomes.  In the fourth paragraph of the ***Discussion***, we replaced deterministic statements such as "will provide early detection" and "will enable clinical decision support" with more cautious wording, including "may serve as a practical tool", "indicates potential for early detection", and "may assist in".  In the seventh paragraph of the ***Discussion*** (the limitations section), we added the following sentence: "...no external validation cohort was available. Future research should establish multicenter prospective cohorts to further support the generalizability of the findings."  ***Supplementary Table S2*** shows that our Ridge model (*AUC* = 0.852) significantly outperformed both the simple disease count model (*AUC* = 0.589) and the age‑sex model (*AUC* = 0.797), demonstrating that our marker provides independent incremental prognostic value in predicting mortality risk.  Thank you again. |

| **Comments** | **Review Comments to the Author** |
| --- | --- |
| **Reviewer # 1** | The evaluation framework also remains unclear. The study first performs a regression task to predict disease count, then turns the predicted score into a classification task. It is still not clear what independent class label is being predicted in the classification analysis. If the class labels are generated from the model output itself, then accuracy, F1 score, AUC, and PR-AUC do not provide meaningful evidence of independent predictive performance. |
| **Response** | The reviewer astutely identified the serious problem of circular classification evaluation in our original manuscript. We fully accept this criticism and express our sincere gratitude for your profound insight. Your guidance has made us realise that a sound evaluation framework must avoid any form of self‑reference. To address this issue, we have completely removed the original classification evaluation framework in the revised manuscript and no longer use the model output itself as classification labels. The new evaluation framework directly assesses the model's ability to predict the probability of death, using standard probabilistic prediction metrics such as *AUC*, *PR‑AUC*, *Brier Score*, and *Log Loss*. All evaluations are performed on an independent test set, thereby completely avoiding circular reasoning. Every piece of guidance you have provided has deepened our understanding of the rigour required in scientific methodology, and we are truly grateful. |
| **Changes in the Paper** | The original ***Table 1*** in our manuscript, which contained *Accuracy, Precision, Recall*, and *F1*, has been completely removed. The original ***Figure 4***, the confusion matrix, has also been deleted. All classification descriptions such as "three‑class classification task" have been removed from the manuscript. We have added a new ***Table 1*** with the title "Predictive performance of machine learning models on the independent test set". This new table only includes *AUC, PR‑AUC, Brier Score, Log Loss, Pseudo R²*, and their respective 95% *Confidence Intervals*. All evaluations were performed on the independent test set.  We have also clarified the evaluation process in ***Methods Section 2.4***. Specifically, we state that the low, middle, and high groups are used only for descriptive analysis (***Table 2***) and do not participate in model performance evaluation. Model performance evaluation uses only probabilistic prediction metrics such as ***AUC***.  The independent test set ensures a circularity‑free evaluation. The test set was completely isolated from data preprocessing, network construction, feature selection, and hyperparameter optimisation. This isolation guarantees that the evaluation results are not contaminated by any training information.  Calibration curve validation further supports the rationality of our evaluation framework. ***Supplementary Figure S1*** presents the calibration curve of the Ridge regression, demonstrating that the predicted probabilities closely agreed with the observed outcomes.  Thank you again. |

| **Comments** | **Review Comments to the Author** |
| --- | --- |
| **Reviewer # 1** | The reported performance metrics require careful rechecking. The RandomForest model shows relatively high accuracy but weak AUC values. In the sensitivity analysis excluding HBALC and glucose, accuracy increases while AUC is approximately 0.50. This pattern does not support robustness. It suggests possible problems with label construction, class imbalance, probability estimation, or AUC calculation. |
| **Response** | We sincerely thank the reviewer for astutely identifying this anomalous finding. Your rigorous attitude made us realise that the counterintuitive result in our original manuscript was actually a manifestation of flaws in the evaluation process. It was precisely your questioning that prompted us to thoroughly reconsider and restructure the entire analytical workflow. In the revised manuscript, because the prediction target has been changed to probability of death and we have adopted an independent test set and nested cross‑validation, the sensitivity issue present in the original manuscript is no longer applicable. We have provided new evidence of robustness. Baseline model comparisons show that performance improved after including key variables, which is consistent with expectations. *Bootstrap* network stability analysis shows that the core edges appeared in over 85% of the replications. Calibration curves show that the predicted probabilities closely agreed with the observed outcomes. Every critical examination you have made has driven us towards higher research standards, and we are deeply grateful. |
| **Changes in the Paper** | The original sensitivity analysis was removed. The paragraph and associated table in the original manuscript regarding the "performance improvement after excluding HBALC and GLU" have been completely deleted.  A new baseline comparison consistent with expectations was added. ***Supplementary Table S2*** shows that M3 (age + sex + basic biochemistry including GLU but not HBALC) achieved an AUC of 0.824, whereas M4 and M5 (including both HBALC and GLU) achieved an AUC of 0.852. This performance improvement demonstrates that HBALC and GLU have positive contributions to predicting mortality risk, which is completely opposite to the counterintuitive result in the original manuscript and aligns with clinical knowledge.  Bootstrap network stability analysis was performed. In ***Methods Section 2.5***, we added a bootstrap analysis with 1,000 resamples and 85% sample size. The core directed edges appeared in at least 85% of the replications. The Kendall's τ coefficients for node centrality rankings were 0.662 (0.077) for degree centrality, 0.461 (0.105) for betweenness centrality, and 0.599 (0.092) for closeness centrality, indicating a robust network structure.  Calibration curve validation was conducted. ***Supplementary Figure S1*** presents the calibration curve of the Ridge model, showing that the predicted probabilities closely agreed with the observed outcomes, thereby supporting the accuracy of probability estimation.  Linearity and non‑Gaussianity validation was performed. In ***Methods Section 2.5***, we added validation for the linearity assumption (residual plots and variance inflation factor) and for non‑Gaussianity (Shapiro‑Wilk test), confirming the suitability of DirectLiNGAM for the present dataset.  Thank you again. |
| **Comments** | **Review Comments to the Author** |
| **Reviewer # 1** | The baseline comparisons are still not strong enough to show added value. The manuscript should compare the proposed approach with simpler and clinically transparent alternatives, including disease count alone, age/sex models, age/sex plus routine laboratory variables, all available laboratory variables, and standard count models. Without these comparisons, it is difficult to judge whether the network-selected marker adds meaningful information. |
| **Response** | We thank the reviewer for this specific and practical suggestion. Your comment has made us deeply realise that systematic baseline comparison is key to demonstrating added value and is an indispensable part of scientific research. It was precisely your request that prompted us to construct five baseline models with increasing complexity, ranging from the simplest simple disease count model to the full variable logistic regression, in order to clearly illustrate the incremental prognostic value of the digital marker proposed in this study. We have learned from this process how to present research findings more objectively and transparently, which will serve as an important model for our future work. Once again, we sincerely thank you for your meticulous guidance. |
| **Changes in the Paper** | A new ***Supplementary Table S2*** was added. Its title is "Predictive performance of five baseline models on the independent test set". The models were defined as follows. M1 was the simple disease count model, the simplest alternative. M2 included age and sex. M3 added basic biochemistry indicators (GLU, TC, UA) to M2. M4 was the full variable logistic regression using all demographic and laboratory variables. M5 was our Ridge model using the key features selected by network analysis.  All evaluation metrics were unified across models. Every model was evaluated on the same independent test set using identical metrics including AUC, PR‑AUC, Brier Score, and Log Loss to ensure fair comparison.  Clear gradient improvement was observed. The results showed a clear gradient from M1 (AUC = 0.589) to M2 (0.797) to M3 (0.824) to M4/M5 (0.852). The PR‑AUC increased from 0.147 for M1 to 0.530 for M5, while the Brier Score decreased from 0.125 to 0.078.  The incremental value of network‑selected features was demonstrated. M4 (full variable logistic regression) and M5 (our Ridge model) achieved nearly identical performance, both with an AUC of 0.852. However, M5 used only seven laboratory features selected by network analysis. This proves that network analysis effectively identified key features, offering advantages in dimensionality reduction and clinical interpretability.  The table was cited and discussed in the main text. It was first cited at the end of ***Results Section 3.4*** and discussed in the fourth paragraph of the ***Discussion***. The text explicitly states that our marker outperforms simple alternatives such as disease count alone.  Thank you again. |

| **Comments** | **Review Comments to the Author** |
| --- | --- |
| **Reviewer # 1** | The reported performance metrics require careful rechecking. The RandomForest model shows relatively high accuracy but weak AUC values. In the sensitivity analysis excluding HBALC and glucose, accuracy increases while AUC is approximately 0.50. This pattern does not support robustness. It suggests possible problems with label construction, class imbalance, probability estimation, or AUC calculation. |
| **Response** | We sincerely thank the reviewer for astutely identifying this anomalous finding. Your rigorous attitude made us realise that the counterintuitive result in our original manuscript was actually a manifestation of flaws in the evaluation process. It was precisely your questioning that prompted us to thoroughly reconsider and restructure the entire analytical workflow. In the revised manuscript, because the prediction target has been changed to probability of death and we have adopted an independent test set and nested cross‑validation, the sensitivity issue present in the original manuscript is no longer applicable. We have provided new evidence of robustness. Baseline model comparisons show that performance improved after including key variables, which is consistent with expectations. *Bootstrap* network stability analysis shows that the core edges appeared in over 85% of the replications. Calibration curves show that the predicted probabilities closely agreed with the observed outcomes. Every critical examination you have made has driven us towards higher research standards, and we are deeply grateful. |
| **Changes in the Paper** | The original sensitivity analysis was removed. The paragraph and associated table in the original manuscript regarding the "performance improvement after excluding HBALC and GLU" have been completely deleted.  A new baseline comparison consistent with expectations was added. ***Supplementary Table S2*** shows that M3 (age + sex + basic biochemistry including GLU but not HBALC) achieved an AUC of 0.824, whereas M4 and M5 (including both HBALC and GLU) achieved an AUC of 0.852. This performance improvement demonstrates that HBALC and GLU have positive contributions to predicting mortality risk, which is completely opposite to the counterintuitive result in the original manuscript and aligns with clinical knowledge.  Bootstrap network stability analysis was performed. In ***Methods Section 2.5***, we added a bootstrap analysis with 1,000 resamples and 85% sample size. The core directed edges appeared in at least 85% of the replications. The Kendall's τ coefficients for node centrality rankings were 0.662 (0.077) for degree centrality, 0.461 (0.105) for betweenness centrality, and 0.599 (0.092) for closeness centrality, indicating a robust network structure.  Calibration curve validation was conducted. ***Supplementary Figure S1*** presents the calibration curve of the Ridge model, showing that the predicted probabilities closely agreed with the observed outcomes, thereby supporting the accuracy of probability estimation.  Linearity and non‑Gaussianity validation was performed. In ***Methods Section 2.5***, we added validation for the linearity assumption (residual plots and variance inflation factor) and for non‑Gaussianity (Shapiro‑Wilk test), confirming the suitability of DirectLiNGAM for the present dataset.  Thank you again. |
| **Comments** | **Review Comments to the Author** |
| **Reviewer # 2** | The revised manuscript shows clear improvements in transparency and statistical reporting… However, several fundamental concerns raised by the reviewers remain insufficiently addressed. Most importantly, the issue of circularity persists: the digital marker is still defined as a predicted disease count and then used to stratify disease burden, raising concerns that the approach largely re-encodes the original outcome rather than providing independent clinical insight. |
| **Response** | We sincerely thank the reviewer for emphasising this fundamental issue once again. Your persistence and rigour have made us deeply realise that circular reasoning is a fatal flaw that must be thoroughly eliminated. It was precisely your repeated reminders that prompted us to unwaveringly shift the prediction target of the digital marker from "disease count" to "probability of death" in the revised manuscript. This change breaks the circular logic because the digital marker no longer predicts its own stratification basis but instead directly estimates an independent hard endpoint, namely mortality risk. At the same time, through baseline model comparisons (***Supplementary Table S2***), we have demonstrated that our marker (*AUC* = 0.852) significantly outperforms the simple disease count model (*AUC* = 0.589), thereby providing independent clinical insight. Every one of your inquiries has deepened our respect for the rigour of scientific research, and we are truly grateful. |
| **Changes in the Paper** | The prediction target was uniformly revised throughout the manuscript. All instances of "predicting disease count" in the ***Abstract, Methods Section 2.4, Results Section 3.4,*** and ***the Discussion*** have been changed to "predicting the probability of death" or "predicting death". The mean values of the digital marker changed from 0.72, 1.56, and 3.02 on the disease count scale to 0.016, 0.058, and 0.296 on the mortality probability scale. This change is clearly presented in Results Section 3.4 and in Table 2.  The low, middle, and high groups were redefined. These three groups now represent subgroups with increasing mortality risk rather than tertiles of disease count. Between‑group comparisons in ***Table 2*** show that the mortality rate in the high group is significantly higher than that in the low group.  A clear comparison was provided in ***Supplementary Table S2***. The simple disease count model (M1) achieved an *AUC* of 0.589, whereas our model (M5) achieved an *AUC* of 0.852, a difference of 0.263. This demonstrates that our marker provides prognostic information independent of disease count.  Calibration was validated. The actual mortality rate in the test set (0.122) closely agreed with the mean predicted probability (0.124), further confirming the accuracy of the digital marker as a measure of mortality risk rather than a mapping of disease count.  Thank you again. |

| **Comments** | **Review Comments to the Author** |
| --- | --- |
| **Reviewer # 2** | Concerns about information leakage also remain unresolved. Although the authors provide conceptual arguments and sensitivity analyses, they do not implement a leakage-safe pipeline (e.g., nested cross-validation), and key steps such as network construction and feature selection appear to be performed on the full dataset. This raises the possibility of optimistic performance estimates. |
| **Response** | The reviewer identified a very serious flaw in our original manuscript. We deeply apologise for this and sincerely thank you for your rigorous scrutiny. Your criticism made us realise that any form of information leakage can lead to overestimated performance and thereby mislead readers. It was precisely your correction that prompted us to implement a strict leakage‑proof pipeline in the revised manuscript. We adopted an independent test set, nested cross validation, and a rigorous procedure in which all preprocessing steps (missing value imputation, standardisation, feature selection) were fitted only on the training subset. This process has given us a deep understanding of the importance of data isolation and has strengthened our confidence in the reproducibility of our findings. Once again, we thank you for your meticulous guidance. |
| **Changes in the Paper** | Independent test set isolation was implemented. In ***Methods Section 2.4***, we added the following text. "Participants were first stratified by their year of enrolment and follow-up. Within each year stratum, a 7:3 random split was performed independently to assign participants to the global training set (Outer Train) and the global test set (Outer Test). The test set was completely excluded from any data preprocessing (including missing value imputation and standardisation), feature selection, causal network construction, and hyperparameter optimisation."  Nested 5×5 cross validation was adopted. The following description was added. "Model training employed a nested 5‑fold cross‑validation framework: the outer 5‑fold loop assessed generalisation performance, while the inner 5‑fold loop performed grid search for hyperparameters. Within each fold, missing value imputation (median), standardisation, and feature selection were independently performed on the training sub‑set and applied to the corresponding validation sub‑set."  Network construction was performed using only the training set. The *DirectLiNGAM* network was constructed solely from the training set, and the test set never participated in any network structure learning. In ***Methods Section 2.5***, the network stability analysis was explicitly marked as being performed "among the train group".  Evaluation results were reported transparently. All performance metrics (***Table 1*** and ***Supplementary Table S2***) were reported based on the independent test set, ensuring the generalisability of the results.Thank you again. |

| **Comments** | **Review Comments to the Author** |
| --- | --- |
| **Reviewer # 2** | Moreover, the sensitivity analysis yields counterintuitive results, with model performance improving after removal of strong predictors (e.g., HBALC and glucose), which raises additional concerns about potential leakage, evaluation inconsistencies, or issues with label definition. |
| **Response** | We thank the reviewer for astutely identifying this counterintuitive result. Your questioning prompted us to deeply reflect on the flaws in the analytical process of our original manuscript. We realised that this anomalous finding was actually a manifestation of the inadequacies in the evaluation framework under the original "predicting disease count" paradigm. In the revised manuscript, because the prediction target has been changed to probability of death and we have adopted an independent test set and nested cross‑validation, we no longer retain the original sensitivity analysis. Instead, we provide new evidence that aligns with expectations. In the baseline model comparisons, model performance improved significantly after including HBALC and GLU, with AUC increasing from 0.824 to 0.852, demonstrating that both indicators have positive predictive value. Every one of your queries has driven us to continuously improve ourselves, and we are truly grateful. |
| **Changes in the Paper** | The original sensitivity analysis was removed. ***Supplementary Table 2*** in the original manuscript, which concerned the exclusion of HBALC and GLU, has been completely deleted, and the related content no longer appears.  New evidence consistent with expectations was added. ***Supplementary Table S2*** shows that M3 (age + sex + basic biochemistry including GLU but not HBALC) achieved an AUC of 0.824, while M4 and M5 (including both HBALC and GLU) achieved an *AUC* of 0.852. This performance improvement aligns with clinical expectations.  Calibration curve validation was performed. ***Supplementary Figure S1*** shows that the predicted probabilities of the Ridge model closely agreed with the observed outcomes, supporting the validity of the label definition.  Network stability analysis was conducted. The bootstrap analysis with 1,000 resamples showed that core edges appeared in at least 85% of the replications, indicating a robust network structure and further supporting the appropriateness of the selected features.  Methodological validation was performed. In ***Methods Section 2.5***, we added validation for the linearity and non‑Gaussianity assumptions to ensure the suitability of DirectLiNGAM.  Thank you again. |

| **Comments** | **Review Comments to the Author** |
| --- | --- |
| **Reviewer # 2** | While the authors have appropriately reframed the study as cross-sectional and softened causal claims, the clinical meaning and utility of the proposed marker remain unclear. The staging framework is still based on arbitrary percentile thresholds without demonstrated linkage to clinically meaningful outcomes. Crucially, no validation against independent endpoints (e.g., hospitalization, cardiovascular events, mortality) is provided, and no external validation is performed. |
| **Response** | The reviewer's concern is entirely reasonable, and we express our sincere gratitude. We acknowledge that our original manuscript had shortcomings in terms of endpoint association and external validation. It was precisely your criticism that prompted us to take the following measures to establish a direct link between the marker and clinical meaning. We set the prediction target as mortality, an independent hard endpoint, and reported the high consistency between the actual mortality rate and the mean predicted probability (0.122 versus 0.124). At the same time, we demonstrated in baseline comparisons that our marker outperforms simple alternatives such as disease count. Regarding the lack of external validation, we have explicitly acknowledged this as a limitation in the manuscript and have identified it as a direction for future research. Your high standards have taught us how to maintain humility and prudence in scientific research, and we are deeply grateful. |
| **Changes in the Paper** | At the end of ***Results Section 3.4***, we added the following sentence. "The actual mortality rate in the test set was 0.122, which was closely approximated by the mean predicted probability (0.124) of the digital marker constructed in this study, indicating good model calibration."  Baseline comparisons demonstrated superiority. ***Supplementary Table S2*** shows that M5 (our Ridge model) achieved an *AUC* of 0.852, which is significantly better than M1 (disease count, *AUC* = 0.589) and M2 (age and sex, *AUC* = 0.797).  A calibration curve was provided. ***Supplementary Figure S1*** shows the agreement between predicted probabilities and actual mortality.  Limitations were explicitly stated. In the seventh paragraph of the Discussion, we added the following sentence. "...no external validation cohort was available. Future research should establish multicenter prospective cohorts to further support the generalizability of the findings."  Robustness of the tertile split was assessed. In ***Methods Section 2.4***, we stated that the split was based on the distribution of the digital marker. We also retained a quartile‑based sensitivity analysis in the supplementary materials (Supplementary Table S4). The results showed a consistent pattern, confirming that the choice of split points did not affect the main conclusions.  Thank you again. |

| **Comments** | **Review Comments to the Author** |
| --- | --- |
| **Reviewer # 3** | Throughout the document there are many errors in linked items (references, chapters and equations) Fig 1, flowchart: blue box on the bottom-right: 1 wave (instead of 1 waves) Line 256: bracket should be deleted Line 260: missing space between “)” and “and” Line 261: missing space between “participants,” and “62.12%” Line 262: typo (“had a formal”) |
| **Response** | We sincerely thank the reviewer for your meticulous proofreading. You took the trouble to point out every formatting error, which made us deeply realise the importance of detail in academic writing. It is precisely your rigour that has taught us that we must scrutinise our manuscript line by line before submission. We sincerely apologise for these oversights and have corrected each one accordingly. Your conscientiousness has deeply touched us and instilled in us a profound respect for academic standards. Once again, we thank you wholeheartedly for your meticulous guidance. |
| **Changes in the Paper** | The specific modifications are as follows. All changes have been marked in red in the Revised Manuscript with Track Changes.  For Figure 1, the text in the bottom box was changed from "1 waves" to "1 wave".  At line 256 (approximately line 258 in the revised manuscript), an extra bracket was deleted.  At line 260 (approximately line 262 in the revised manuscript), a missing space was added between ")" and "and", resulting in ") and".  At line 261 (approximately line 263 in the revised manuscript), a missing space was added after "participants,", resulting in "participants, 62.12%".  At line 262, the typo "had a formal schooling" was corrected to "had formal schooling".  Thank you again. |

| **Comments** | **Review Comments to the Author** |
| --- | --- |
| **Reviewer # 3** | Table 2 could be shortened considerably if only one category would be shown for all binary variables. For example, for marital status, it would be enough to just write “Marital status, reference=unmarried" and then only show the married (or vice-versa). The same applies to the other binary variables. And what is the motivation to show both t/r and p-value? P-value would be sufficient. I also suggest rounding all values to 1 decimal place (except for the p-value). Instead of writing “History of” you could add a separate row “Medical history” and use indention for all the diseases. The abbreviations should be explained in the table legend. |
| **Response** | Thank you for these very practical suggestions for improving the tables. Your comments have made us realise that tables are not only a means of presenting data but also an important window for readers to quickly understand the research findings. We have fully adopted your suggestions and thoroughly streamlined and restructured Table 2. This process has taught us how to design clearer and more reader‑friendly displays from the perspective of the audience. We have greatly benefited from every piece of guidance you have provided, and we are truly grateful.    Thank you again. |

| **Comments** | **Review Comments to the Author** |
| --- | --- |
| **Reviewer # 3** | For Table 3 I suggest to only show the estimate and corresponding 95% CI and p-value. SE and t do not contribute any information if CI and p-value are shown. It might be helpful to add a forest plot to this table (which show the estimate and 95%CI). Again, the abbreviations should be in the table legend. |
| **Response** | Thank you for your suggestions regarding the conciseness of tables. Your comments have made us realise that statistical tables should present only the most essential information and avoid redundancy. We have modified Table 3 as requested and added a forest plot to visually display the effect estimates of each variable. Every suggestion you have made has deepened our understanding of data visualisation, and we are truly grateful.      Thank you again. |
