# Supplementary material for: A digital marker for stratifying cardiovascular metabolic comorbidities among the middle-aged and elderly adults
Source: PLOS Digit Health. 2026 Jul 2;5(7):e0001528. doi: 10.1371/journal.pdig.0001528 (PMC13327254; doi:10.1371/journal.pdig.0001528)
Supplement: Peer review history

# Peer review: Decision Letter 0

**DOI:** 10.1371/journal.pdig.0001528.r001

**Authors:** Dhiya Al-Jumeily OBE, Shrey Lakhotia

12 Mar 2026

PDIG-D-26-00009

A Digital Marker for Staging Cardiovascular Metabolic Comorbidities among the Middle-Aged and Elderly Adults

PLOS Digital Health

Dear Dr. Mao,

Thank you for submitting your manuscript to PLOS Digital Health. After careful consideration, we feel that it has merit but does not fully meet PLOS Digital Health's publication criteria as it currently stands. Therefore, we invite you to submit a revised version of the manuscript that addresses the points raised during the review process.

Please submit your revised manuscript by May 11 2026 11:59PM. If you will need more time than this to complete your revisions, please reply to this message or contact the journal office at digitalhealth@plos.org. When you're ready to submit your revision, log on to https://www.editorialmanager.com/pdig/ and select the 'Submissions Needing Revision' folder to locate your manuscript file.

Please include the following items when submitting your revised manuscript:

* A letter that responds to each point raised by the editor and reviewer(s). You should upload this letter as a separate file labeled 'Response to Reviewers'. This file does not need to include responses to any formatting updates and technical items listed in the 'Journal Requirements' section below.

* A marked-up copy of your manuscript that highlights changes made to the original version. You should upload this as a separate file labeled 'Revised Manuscript with Track Changes'.

* An unmarked version of your revised paper without tracked changes. You should upload this as a separate file labeled 'Manuscript'.

If you would like to make changes to your financial disclosure, competing interests statement, or data availability statement, please make these updates within the submission form at the time of resubmission. Guidelines for resubmitting your figure files are available below the reviewer comments at the end of this letter.

We look forward to receiving your revised manuscript.

Kind regards,

Shrey Lakhotia

Academic Editor

PLOS Digital Health

Dhiya Al-Jumeily OBE

Section Editor

PLOS Digital Health

Leo Anthony Celi

Editor-in-Chief

PLOS Digital Health

orcid.org/0000-0001-6712-6626

**Journal Requirements:**

1. Please amend your detailed Financial Disclosure statement. This is published with the article. It must therefore be completed in full sentences and contain the exact wording you wish to be published.

i. Please clarify all sources of financial support for your study. List the grants, grant numbers, and organizations that funded your study, including funding received from your institution. Please note that suppliers of material support, including research materials, should be recognized in the Acknowledgements section rather than in the Financial Disclosure.

ii. State the initials, alongside each funding source, of each author to receive each grant. For example: "This work was supported by the National Institutes of Health (####### to AM; ###### to CJ) and the National Science Foundation (###### to AM)."

iii. State what role the funders took in the study. If the funders had no role in your study, please state: “The funders had no role in study design, data collection and analysis, decision to publish, or preparation of the manuscript.”

iv. If any authors received a salary from any of your funders, please state which authors and which funders.

2. Please ensure that your Ethics Statement is available in its entirety at the beginning of your Methods section, under a subheading 'Ethics Statement'.

3. We ask that a manuscript source file is provided at Revision. Please upload your manuscript file as a .doc, .docx, .rtf or .tex.

4. Please upload separate figure files in .tif or .eps format. Also, remove the figures from your manuscript file but keep the legends.

For more information about figure files please see our guidelines:

https://journals.plos.org/digitalhealth/s/figures

https://journals.plos.org/digitalhealth/s/figures#loc-file-requirements

5. Please provide an Author Summary. This should appear in your manuscript between the Abstract (if applicable) and the Introduction, and should be 150–200 words long. The aim should be to make your findings accessible to a wide audience that includes both scientists and non-scientists. Sample summaries can be found on our website under Submission Guidelines:

https://journals.plos.org/digitalhealth/s/submission-guidelines#loc-parts-of-a-submission

If the reviewer comments include a recommendation to cite specific previously published works, please review and evaluate these publications to determine whether they are relevant and should be cited. There is no requirement to cite these works unless the editor has indicated otherwise.

**Additional Editor Comments (if provided):**

The manuscript addresses a clinically relevant topic regarding the use of computational methods and digital markers for health risk stratification. However, as noted by two reviewers, the statistical analysis is not sufficiently rigorous, and there are fundamental methodological concerns (circularity, information leakage, mismatch between study design and claims) that prevent the manuscript from meeting the journal’s publication criteria in its current form.

[Note: HTML markup is below. Please do not edit.]

**Reviewers' Comments:**

Reviewer's Responses to Questions

**Comments to the Author**

1. Does this manuscript meet PLOS Digital Health’s publication criteria? Is the manuscript technically sound, and do the data support the conclusions? The manuscript must describe methodologically and ethically rigorous research with conclusions that are appropriately drawn based on the data presented.

Reviewer #1: Partly

Reviewer #2: Yes

Reviewer #3: No

**********

2. Has the statistical analysis been performed appropriately and rigorously?

Reviewer #1: No

Reviewer #2: Yes

Reviewer #3: No

**********

3. Have the authors made all data underlying the findings in their manuscript fully available (please refer to the Data Availability Statement at the start of the manuscript PDF file)?

The PLOS Data policy requires authors to make all data underlying the findings described in their manuscript fully available without restriction, with rare exception. The data should be provided as part of the manuscript or its supporting information, or deposited to a public repository. For example, in addition to summary statistics, the data points behind means, medians and variance measures should be available. If there are restrictions on publicly sharing data—e.g. participant privacy or use of data from a third party—those must be specified.

Reviewer #1: Yes

Reviewer #2: Yes

Reviewer #3: Yes

**********

4. Is the manuscript presented in an intelligible fashion and written in standard English?

PLOS Digital Health does not copyedit accepted manuscripts, so the language in submitted articles must be clear, correct, and unambiguous. Any typographical or grammatical errors should be corrected at revision, so please note any specific errors here.

Reviewer #1: Yes

Reviewer #2: Yes

Reviewer #3: No

**********

5. Review Comments to the Author

Please use the space provided to explain your answers to the questions above. You may also include additional comments for the author, including concerns about dual publication, research ethics, or publication ethics. (Please upload your review as an attachment if it exceeds 20,000 characters)

**Reviewer #1:**  Thank you for the submission. The idea—building a digital marker to help stage cardiovascular–metabolic multimorbidity (CMM) in middle-aged and older adults—fits the scope of PLOS Digital Health and could be clinically useful. That said, I recommend Major Revision because several “foundation-level” items are currently not clear enough (or not validated strongly enough) to support the manuscript’s main claims. In plain terms: right now I cannot tell (i) what the proposed “stages” truly mean clinically, and (ii) whether the modeling and evaluation are set up in a way that rules out shortcuts, leakage, or over-interpretation.

1) What exactly is being “staged,” and is it more than a renamed disease count?

As written, the workflow appears to train models to predict disease count and then use the resulting marker to assign Early/Middle/Late stages. This risks circularity: if “stage” is derived from disease count thresholds (directly or indirectly), then the marker may simply reproduce the same information in a more complex form. Please define clearly: (a) the exact diseases included in “disease count,” diagnostic criteria and time window; (b) how Early/Middle/Late labels are created (fixed cutoffs? data-driven clustering? clinical rationale?); and (c) why these stages reflect clinically meaningful progression rather than a convenient binning. Critically, please demonstrate added value beyond simple disease count stratification—for example by linking stages to independent outcomes (future disease accrual, hospitalization, cardiovascular events, mortality) or other clinically accepted endpoints.

2) The prediction task and the reported evaluation metrics do not line up (regression vs classification).

If the primary target is a count (disease count), the natural framing is regression or count modeling; however, the reported metrics (Accuracy/Precision/Recall/F1) are classification metrics. Please clarify the primary endpoint and align the analysis accordingly. If you are doing regression, report MAE/RMSE/R² (or count-model metrics such as Poisson/negative binomial where appropriate). If you are doing classification (e.g., stage classification), specify class definitions and prevalence, report appropriate discrimination metrics (e.g., AUC/PR-AUC as relevant), and include calibration. In either case, provide uncertainty (confidence intervals) via bootstrap or repeated/nested cross-validation rather than a single split.

3) High risk of information leakage / “shortcut learning” needs to be explicitly addressed.

Given that key lab markers such as HbA1c/GLU are closely tied to diabetes (and diabetes appears among disease nodes), the model could achieve strong performance by detecting an obvious disease proxy rather than capturing a generalizable multimorbidity staging signal. In addition, the overall pipeline (network construction → feature ranking/selection → model training) is prone to leakage if any step uses the full dataset before train/test separation. Please document a leakage-safe procedure: network learning and feature selection must occur within training folds only (nested CV), not on the entire dataset. I also strongly recommend sensitivity analyses such as: removing high-leakage proxy features (e.g., HbA1c/GLU) and reporting performance drop; or redefining the outcome (if clinically justified) to show the marker is not dominated by a single disease proxy.

4) The causal discovery / DAG component (DirectLiNGAM) requires stronger justification and robustness checks.

DirectLiNGAM relies on assumptions (e.g., linear relations, non-Gaussian noise, acyclicity, limited unmeasured confounding) that can be difficult to satisfy in mixed clinical data, especially when combining continuous labs with binary disease indicators. Please provide enough methodological detail for readers to assess appropriateness: variable types/encoding, preprocessing, missing data handling, and any assumption checks. Also, the results should be shown to be stable: edge stability and centrality ranking stability via bootstrap/resampling; sensitivity to preprocessing; and ideally comparison to at least one alternative structure-learning approach. Finally, please use careful language: unless assumptions are convincingly supported, the DAG/edges should be described as structural/associational rather than definitive causal mechanisms.

5) Claims about “dynamic evolution/progression” must match the study design.

If the study is cross-sectional, it cannot directly demonstrate progression over time; in that case, the manuscript should be reframed as cross-sectional stratification. If longitudinal data exist, please explicitly describe the time structure and validate the “stages” by showing transitions or prediction of future disease accumulation, ideally with out-of-time validation.

6) Clinical usefulness is not yet demonstrated; baseline comparisons and validation are needed.

Even if the model predicts disease count well, readers need to know whether it improves on simpler alternatives (age/sex + basic labs; standard statistical models; direct disease-count thresholds). Please add strong baselines and show incremental value. External validation is preferred; if not possible, at least provide rigorous internal validation (nested CV, or time-split validation when appropriate). Consider adding calibration and decision-analytic evaluation (e.g., decision curve analysis) if you want to claim clinical decision support value.

In summary, the manuscript has a potentially valuable direction, but the current version leaves key questions unanswered: what the “stages” mean clinically, whether the evaluation is leakage-safe and appropriately measured, whether the causal/network claims are robust, and whether the marker offers real added value beyond disease count. Addressing the above points with clear definitions, rigorous validation, and reproducible reporting would substantially improve technical soundness and the credibility of the conclusions.

**Reviewer #2:**  Overall, the study addresses a clinically relevant and timely topic aligned with the scope of PLOS Digital Health, and the methodological framework is generally sound.

The rationale for categorizing the continuous digital marker into three stages using tertile-based cut-points (P33 and P67) requires further clarification. While this approach is statistically convenient, the manuscript would benefit from a clearer explanation of how these thresholds relate to clinically meaningful distinctions in cardiometabolic multimorbidity progression. In addition, the robustness of the proposed staging framework could be strengthened by conducting sensitivity analyses using alternative cut-points (e.g., quartiles or clinically informed thresholds) to demonstrate that the observed patterns and conclusions are not highly dependent on the specific binning strategy chosen.

Although the reported model performance metrics (accuracy, precision, recall, and F1 score) are acceptable for comparing algorithms, the absence of calibration assessment limits evaluation of the model’s clinical applicability. Reporting calibration measures, such as calibration plots, would provide important insight into how well predicted disease burden aligns with observed outcomes across the range of the digital marker. Including such analyses would enhance the interpretability and potential translational value of the proposed digital marker in real-world clinical or population health settings.

**Reviewer #3:**  General comment:

The manuscript is difficult to follow in its current form. The overall purpose of the paper is not sufficiently clear. It remains ambiguous whether the authors aim to present a methodological study (as suggested in the discussion) or to emphasize the clinical relevance of their analysis (as stated in the introduction). I recommend clearly focusing on one of these objectives.

The manuscript would benefit from more streamlined writing. At present, methodological details appear in the background section, while the discussion does not sufficiently elaborate on the clinical implications of the findings. In addition, the results presented do not fully support the conclusions drawn.

Major comments:

Introduction

The first sentence raises several questions. It may not be obvious to readers why CMM is described as a complex disease, nor why there is such a marked difference in prevalence between China and the Middle East/North Africa. What about other regions of the world, and what is the rationale for focusing exclusively on these areas? More generally, I suggest revising the opening section of the introduction, as it currently presents a sequence of facts without a clear narrative or defined objective.

In the final paragraph of the introduction, I recommend focusing more explicitly on the study objectives and moving detailed methodological information to the Methods section.

Methods

What is the rationale for comparing ten different machine learning models?

How were repeated measurements handled for patients who contributed data from two waves?

Results

There are too many figures and tables, and several are difficult to read without magnification. I recommend selecting a smaller number of key figures and summarizing the remaining results in the text. For example, Figure 4 adds limited value in its current form. The heatmap is difficult to interpret, and without sufficient explanation, its purpose remains unclear.

Regarding Table 2, I do not understand the rationale for presenting relative frequencies within categories of a single variable. Would it not be more informative to present relative frequencies across all stages?

Given the sample size, statistically significant associations in multiple linear regression are not unexpected. However, it remains unclear whether the observed effects are clinically meaningful.

Discussion

I did not gain the impression that the paper clearly identifies the core driving factors underlying the evolution of the CMM network. The clinical message should be articulated more clearly.

In addition, the discussion repeats several aspects of the results across different sections. I suggest first discussing the main findings concisely and then placing them into a broader context. This would help distinguish which points stem directly from your results and which are supported by the existing literature.

Minor comments:

Line 97: Missing space between two words.

Figure 1: What is the rationale for displaying boxes for non-existent participants with more than two waves of data? Additionally, why does the top box state “with 5 fives,” which does not appear to be correct for the analyzed sample?

Lines 129–131: Please add a reference.

Line 179: XGBoost is mentioned twice.

Line 182: How were the thresholds determined?

Line 183: Why were five nearest neighbours chosen?

Line 201: Missing space between two words.

Line 202: Missing space between two words.

Line 209: Consider rephrasing to “included in this analysis.”

Line 210ff: I suggest rounding all numbers to one decimal place.

Line 225: Use “married” instead of “Marry.”

Table 2: For binary variables, consider presenting only one category. Also, instead of “didn’t have”/“have,” use “history of …”

Lines 294 and 304: Is this statement based on your results or on the literature? Please clarify the source and provide a reference if applicable.

**********

6. PLOS authors have the option to publish the peer review history of their article (what does this mean?). If published, this will include your full peer review and any attached files.

**Do you want your identity to be public for this peer review?**  If you choose “no”, your identity will remain anonymous but your review may still be made public.

For information about this choice, including consent withdrawal, please see our Privacy Policy.

Reviewer #1: No

Reviewer #2: No

Reviewer #3: No

**********

[NOTE: If reviewer comments were submitted as an attachment file, they will be attached to this email and accessible via the submission site. Please log into your account, locate the manuscript record, and check for the action link "View Attachments". If this link does not appear, there are no attachment files.]

**Figure resubmission:**

While revising your submission, we strongly recommend that you use PLOS’s NAAS tool (https://ngplosjournals.pagemajik.ai/artanalysis) to test your figure files. NAAS can convert your figure files to the TIFF file type and meet basic requirements (such as print size, resolution), or provide you with a report on issues that do not meet our requirements and that NAAS cannot fix.

After uploading your figures to PLOS’s NAAS tool - https://ngplosjournals.pagemajik.ai/artanalysis, NAAS will process the files provided and display the results in the "Uploaded Files" section of the page as the processing is complete. If the uploaded figures meet our requirements (or NAAS is able to fix the files to meet our requirements), the figure will be marked as "fixed" above. If NAAS is unable to fix the files, a red "failed" label will appear above. When NAAS has confirmed that the figure files meet our requirements, please download the file via the download option, and include these NAAS processed figure files when submitting your revised manuscript.

**Reproducibility:**

To enhance the reproducibility of your results, we recommend that authors of applicable studies deposit laboratory protocols in protocols.io, where a protocol can be assigned its own identifier (DOI) such that it can be cited independently in the future. Additionally, PLOS ONE offers an option to publish peer-reviewed clinical study protocols. Read more information on sharing protocols at https://plos.org/protocols?utm_medium=editorial-email&utm_source=authorletters&utm_campaign=protocols

# Author comment: Author response to Decision Letter 1

**DOI:** 10.1371/journal.pdig.0001528.r002

28 Apr 2026

Attachment Submitted filename: renamed_88607.docx

# Peer review: Decision Letter 1

**DOI:** 10.1371/journal.pdig.0001528.r003

**Authors:** Dhiya Al-Jumeily OBE, Shrey Lakhotia, Dhiya Al-Jumeily OBE

26 May 2026

PDIG-D-26-00009R1

A Digital Marker for Stratifying Cardiovascular Metabolic Comorbidities among the Middle-Aged and Elderly Adults

PLOS Digital Health

Dear Dr. Mao,

Thank you for submitting your manuscript to PLOS Digital Health. After careful consideration, we feel that it has merit but does not fully meet PLOS Digital Health's publication criteria as it currently stands. Therefore, we invite you to submit a revised version of the manuscript that addresses the points raised during the review process.

Please submit your revised manuscript by Jul 25 2026 11:59PM. If you will need more time than this to complete your revisions, please reply to this message or contact the journal office at digitalhealth@plos.org. When you're ready to submit your revision, log on to https://www.editorialmanager.com/pdig/ and select the 'Submissions Needing Revision' folder to locate your manuscript file.

Please include the following items when submitting your revised manuscript:

* A letter that responds to each point raised by the editor and reviewer(s). You should upload this letter as a separate file labeled 'Response to Reviewers'. This file does not need to include responses to any formatting updates and technical items listed in the 'Journal Requirements' section below.

* A marked-up copy of your manuscript that highlights changes made to the original version. You should upload this as a separate file labeled 'Revised Manuscript with Track Changes'.

* An unmarked version of your revised paper without tracked changes. You should upload this as a separate file labeled 'Manuscript'.

If you would like to make changes to your financial disclosure, competing interests statement, or data availability statement, please make these updates within the submission form at the time of resubmission. Guidelines for resubmitting your figure files are available below the reviewer comments at the end of this letter.

As the corresponding author, your ORCID iD is verified in the submission system and will appear in the published article. PLOS supports the use of ORCID, and we encourage all coauthors to register for an ORCID iD and use it as well. Please encourage your coauthors to verify their ORCID iD within the submission system before final acceptance, as unverified ORCID iDs will not appear in the published article. Only the individual author can complete the verification step; PLOS staff cannot verify ORCID iDs on behalf of authors.

We look forward to receiving your revised manuscript.

Kind regards,

Dhiya Al-Jumeily OBE, PhD

Section Editor

PLOS Digital Health

Leo Anthony Celi

Editor-in-Chief

PLOS Digital Health

orcid.org/0000-0001-6712-6626

**Journal Requirements:**

1. Please ensure that your Ethics Statement is available in its entirety at the beginning of your Methods section, under a subheading 'Ethics Statement'. It must include:

i) The name(s) of the Institutional Review Board(s) or Ethics Committee(s)

ii) The approval number(s), or a statement that approval was granted by the named board(s)

iii) (for human participants or donors) - A statement that formal consent was obtained (must state whether verbal/written) OR the reason consent was not obtained (e.g., anonymity).

vi) (for child participants) - Please update your Ethics Statement with confirmation that written informed consent was obtained from the parent/guardian of each participant under 18 years of age.

If the reviewer comments include a recommendation to cite specific previously published works, please review and evaluate these publications to determine whether they are relevant and should be cited. There is no requirement to cite these works unless the editor has indicated otherwise.

**Additional Editor Comments (if provided):**

[Note: HTML markup is below. Please do not edit.]

**Reviewers' Comments:**

Reviewer's Responses to Questions

**Comments to the Author**

1. If the authors have adequately addressed your comments raised in a previous round of review and you feel that this manuscript is now acceptable for publication, you may indicate that here to bypass the “Comments to the Author” section, enter your conflict of interest statement in the “Confidential to Editor” section, and submit your "Accept" recommendation.

Reviewer #1: All comments have been addressed

Reviewer #2: (No Response)

Reviewer #3: (No Response)

**********

2. Does this manuscript meet PLOS Digital Health’s publication criteria? Is the manuscript technically sound, and do the data support the conclusions? The manuscript must describe methodologically and ethically rigorous research with conclusions that are appropriately drawn based on the data presented.

Reviewer #1: No

Reviewer #2: Partly

Reviewer #3: Partly

**********

3. Has the statistical analysis been performed appropriately and rigorously?

Reviewer #1: No

Reviewer #2: Yes

Reviewer #3: Yes

**********

4. Have the authors made all data underlying the findings in their manuscript fully available (please refer to the Data Availability Statement at the start of the manuscript PDF file)?

The PLOS Data policy requires authors to make all data underlying the findings described in their manuscript fully available without restriction, with rare exception. The data should be provided as part of the manuscript or its supporting information, or deposited to a public repository. For example, in addition to summary statistics, the data points behind means, medians and variance measures should be available. If there are restrictions on publicly sharing data—e.g. participant privacy or use of data from a third party—those must be specified.

Reviewer #1: Yes

Reviewer #2: Yes

Reviewer #3: Yes

**********

5. Is the manuscript presented in an intelligible fashion and written in standard English?

PLOS Digital Health does not copyedit accepted manuscripts, so the language in submitted articles must be clear, correct, and unambiguous. Any typographical or grammatical errors should be corrected at revision, so please note any specific errors here.

Reviewer #1: Yes

Reviewer #2: Yes

Reviewer #3: No

**********

6. Review Comments to the Author

Please use the space provided to explain your answers to the questions above. You may also include additional comments for the author, including concerns about dual publication, research ethics, or publication ethics. (Please upload your review as an attachment if it exceeds 20,000 characters)

**Reviewer #1:**  The revision has improved the manuscript in wording and presentation, but the main methodological concerns remain unresolved. I do not think the manuscript is ready for publication in its current form.

My main concern is that the proposed digital marker is still too closely tied to disease count. The marker is defined as the disease count predicted by the machine learning model, and the Low/Middle/High groups are then created by splitting this predicted marker into tertiles. This does not yet show that the marker captures a clinically meaningful state beyond disease enumeration. It remains closer to a disease-count prediction and binning exercise than to an independently validated clinical stratification tool.

The manuscript also still lacks independent validation of clinical value. The authors state that future studies may evaluate hospitalization, cardiovascular events, and mortality. Those are exactly the outcomes needed to support the proposed clinical use of the marker. Without such validation, claims about early identification, clinical decision support, and subgroup-based intervention should be substantially reduced.

The evaluation framework also remains unclear. The study first performs a regression task to predict disease count, then turns the predicted score into a classification task. It is still not clear what independent class label is being predicted in the classification analysis. If the class labels are generated from the model output itself, then accuracy, F1 score, AUC, and PR-AUC do not provide meaningful evidence of independent predictive performance.

The reported performance metrics require careful rechecking. The RandomForest model shows relatively high accuracy but weak AUC values. In the sensitivity analysis excluding HbA1c and glucose, accuracy increases while AUC is approximately 0.50. This pattern does not support robustness. It suggests possible problems with label construction, class imbalance, probability estimation, or AUC calculation.

The baseline comparisons are still not strong enough to show added value. The manuscript should compare the proposed approach with simpler and clinically transparent alternatives, including disease count alone, age/sex models, age/sex plus routine laboratory variables, all available laboratory variables, and standard count models. Without these comparisons, it is difficult to judge whether the network-selected marker adds meaningful information.

In summary, the revision addresses some surface-level issues but does not resolve the central problems: circularity, unclear classification evaluation, lack of leakage-safe validation, weak evidence of added clinical value, and over-interpretation of network findings. I recommend major revision.

**Reviewer #2:**  The revised manuscript shows clear improvements in transparency and statistical reporting, particularly in clarifying the two-step modeling framework, adding appropriate regression and classification metrics, and moderating causal language. However, several fundamental concerns raised by the reviewers remain insufficiently addressed.

Most importantly, the issue of circularity persists: the digital marker is still defined as a predicted disease count and then used to stratify disease burden, raising concerns that the approach largely re-encodes the original outcome rather than providing independent clinical insight. Relatedly, while evaluation metrics have been expanded, the classification task is derived directly from the same predicted quantity, limiting its interpretability and added value.

Concerns about information leakage also remain unresolved. Although the authors provide conceptual arguments and sensitivity analyses, they do not implement a leakage-safe pipeline (e.g., nested cross-validation), and key steps such as network construction and feature selection appear to be performed on the full dataset. This raises the possibility of optimistic performance estimates. Moreover, the sensitivity analysis yields counterintuitive results, with model performance improving after removal of strong predictors (e.g., HbA1c and glucose), which raises additional concerns about potential leakage, evaluation inconsistencies, or issues with label definition. This further underscores the need for clarification on whether all preprocessing steps were confined within training folds and whether strict separation between training and test data was maintained throughout.

While the authors have appropriately reframed the study as cross-sectional and softened causal claims, the clinical meaning and utility of the proposed marker remain unclear. The staging framework is still based on arbitrary percentile thresholds without demonstrated linkage to clinically meaningful outcomes. Crucially, no validation against independent endpoints (e.g., hospitalization, cardiovascular events, mortality) is provided, and no external validation is performed. As a result, it remains uncertain whether the proposed digital marker offers meaningful improvement over simpler alternatives such as disease count or basic clinical models.

**Reviewer #3:**  The authors have clearly invested substantial effort into revising the manuscript, and the overall quality has improved considerably compared with the original version. The revisions have strengthened several aspects of the work. However, despite these improvements, there remain major issues that need to be addressed before the manuscript can be considered for publication.

Minor comments

Throughout the document there are many errors in linked items (references, chapters and equations)

Fig 1, flowchart: blue box on the bottom-right: 1 wave (instead of 1 waves)

Line 256: bracket should be deleted

Line 260: missing space between “)” and “and”

Line 261: missing space between “participants,” and “62.12%”

Line 262: typo (“had a formal”)

Major comments

I still find the display items very difficult to read and overwhelming.

Table 2 could be shortened considerably if only one category would be shown for all binary variables. For example, for marital status, it would be enough to just write “Marital status, reference=unmarried" and then only show the married (or vice-versa). The same applies to the other binary variables. And what is the motivation to show both t/r and p-value? P-value would be sufficient. I also suggest rounding all values to 1 decimal place (except for the p-value). Instead of writing “History of” you could add a separate row “Medical history” and use indention for all the diseases. The abbreviations should be explained in the table legend.

For Table 3 I suggest to only show the estimate and corresponding 95% CI and p-value. SE and t do not contribute any information if CI and p-value are shown. It might be helpful to add a forest plot to this table (which show the estimate and 95%CI). Again, the abbreviations should be in the table legend.

**********

7. PLOS authors have the option to publish the peer review history of their article (what does this mean?). If published, this will include your full peer review and any attached files.

**Do you want your identity to be public for this peer review?**  If you choose “no”, your identity will remain anonymous but your review may still be made public.

For information about this choice, including consent withdrawal, please see our Privacy Policy.

Reviewer #1: No

Reviewer #2: No

Reviewer #3: No

**********

[NOTE: If reviewer comments were submitted as an attachment file, they will be attached to this email and accessible via the submission site. Please log into your account, locate the manuscript record, and check for the action link "View Attachments". If this link does not appear, there are no attachment files.]

**Figure resubmission:**

While revising your submission, we strongly recommend that you use PLOS’s NAAS tool (https://ngplosjournals.pagemajik.ai/artanalysis) to test your figure files. NAAS can convert your figure files to the TIFF file type and meet basic requirements (such as print size, resolution), or provide you with a report on issues that do not meet our requirements and that NAAS cannot fix.

After uploading your figures to PLOS’s NAAS tool - https://ngplosjournals.pagemajik.ai/artanalysis, NAAS will process the files provided and display the results in the "Uploaded Files" section of the page as the processing is complete. If the uploaded figures meet our requirements (or NAAS is able to fix the files to meet our requirements), the figure will be marked as "fixed" above. If NAAS is unable to fix the files, a red "failed" label will appear above. When NAAS has confirmed that the figure files meet our requirements, please download the file via the download option, and include these NAAS processed figure files when submitting your revised manuscript.

**Reproducibility:**

To enhance the reproducibility of your results, we recommend that authors of applicable studies deposit laboratory protocols in protocols.io, where a protocol can be assigned its own identifier (DOI) such that it can be cited independently in the future. Additionally, PLOS ONE offers an option to publish peer-reviewed clinical study protocols. Read more information on sharing protocols at https://plos.org/protocols?utm_medium=editorial-email&utm_source=authorletters&utm_campaign=protocols

# Author comment: Author response to Decision Letter 2

**DOI:** 10.1371/journal.pdig.0001528.r004

8 Jun 2026

Attachment Submitted filename: renamed_8a8b9.docx

# Editor report: Decision Letter 2

**DOI:** 10.1371/journal.pdig.0001528.r005

**Authors:** Dhiya Al-Jumeily OBE, Shrey Lakhotia, Dhiya Al-Jumeily OBE, Dhiya Al-Jumeily OBE

13 Jun 2026

A Digital Marker for Stratifying Cardiovascular Metabolic Comorbidities among the Middle-Aged and Elderly Adults

PDIG-D-26-00009R2

Dear Dr. Mao,

We are pleased to inform you that your manuscript 'A Digital Marker for Stratifying Cardiovascular Metabolic Comorbidities among the Middle-Aged and Elderly Adults' has been provisionally accepted for publication in PLOS Digital Health.

Before your manuscript can be formally accepted you will need to complete some formatting changes, which you will receive in a follow-up email from a member of our team.

Please note that your manuscript will not be scheduled for publication until you have made the required changes, so a swift response is appreciated.

IMPORTANT: The editorial review process is now complete. PLOS will only permit corrections to spelling, formatting or significant scientific errors from this point onwards. Requests for major changes, or any which affect the scientific understanding of your work, will cause delays to the publication date of your manuscript.

If your institution or institutions have a press office, please notify them about your upcoming paper to help maximize its impact. If they'll be preparing press materials, please inform our press team as soon as possible -- no later than 48 hours after receiving the formal acceptance. Your manuscript will remain under strict press embargo until 2 pm Eastern Time on the date of publication. For more information, please contact digitalhealth@plos.org.

Thank you again for supporting Open Access publishing; we are looking forward to publishing your work in PLOS Digital Health.

Best regards,

Dhiya Al-Jumeily OBE, PhD

Section Editor

PLOS Digital Health

***********************************************************

**Additional Editor Comments (if provided):**

**Reviewer Comments (if any, and for reference):**
